# Supplementary material for: Top-down coordination of local cortical state during selective attention
Source: Neuron. 2021 Mar 3;109(5):894–904.e8. doi: 10.1016/j.neuron.2020.12.013 (PMC7927916; doi:10.1016/j.neuron.2020.12.013)
Supplement: Document S1. Figures S1–S6 and Table S1 [file mmc1.pdf]

Neuron, Volume *109*

Supplemental information

Top-down coordination of local cortical state during selective attention

Jochem van Kempen, Marc A. Gieselmann, Michael Boyd, Nicholas A. Steinmetz, Tirin Moore, Tatiana A. Engel, and Alexander Thiele

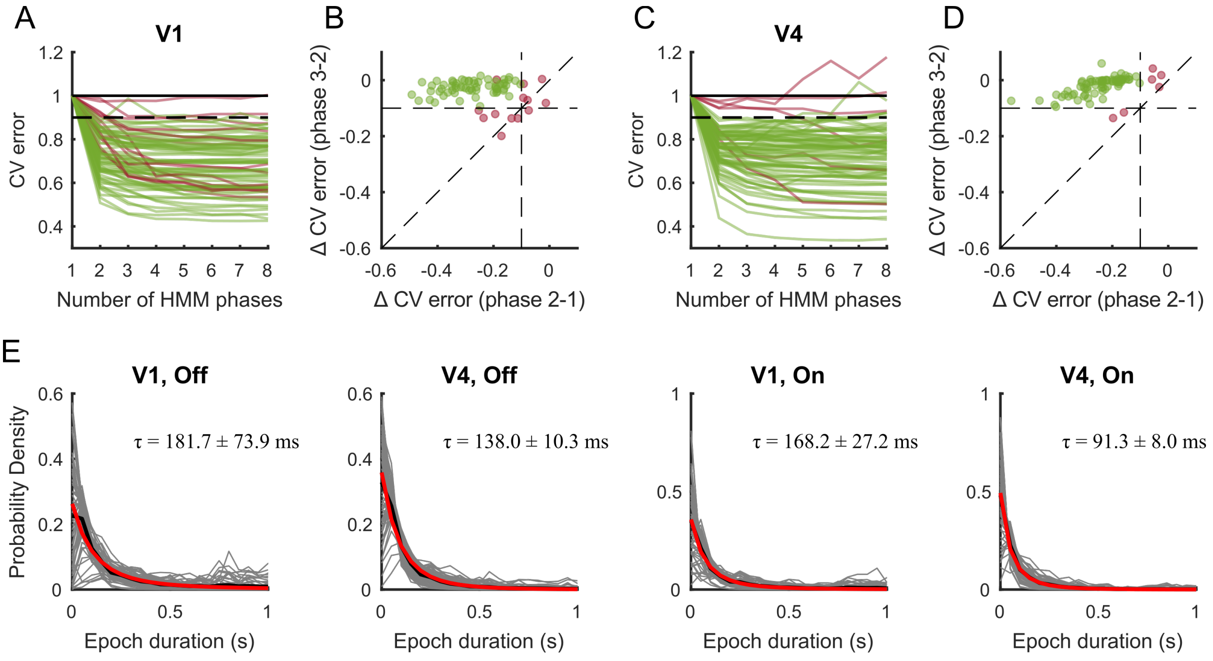

Figure S1. Determining the number of HMM phases and their epoch durations in V1 and V4 MUA, related to STAR Methods. (A) Cross validation (CV) error plotted against the number of phases in each HMM for V1. (B) The difference in cross validation error between the 1-phase and 2-phase model, plotted against the difference between the 2-phase and 3-phase model for V1. Most recordings show a large reduction in cross-validation error with the addition of a second phase and only marginal changes with additional phases. Green (red) lines and markers indicate the recordings included (excluded) for further analysis. (C-D) Same conventions as (A-B) but for V4. (E) Distributions of Off and On episode durations overlaid by the exponential distributions with the decay constant set by the HMM transition probabilities (red, $N(t) = N_0 e^{-t/\tau}$, where N_0 is the normalisation constant, and τ is the decay time-constant computed for each recording and phase). A good match for these models indicates that On-Off dynamics were not driven by an oscillatory phenomenon. Grey and thick black lines indicate individual recordings and their mean, respectively. Data are represented as mean ± SEM across recordings.

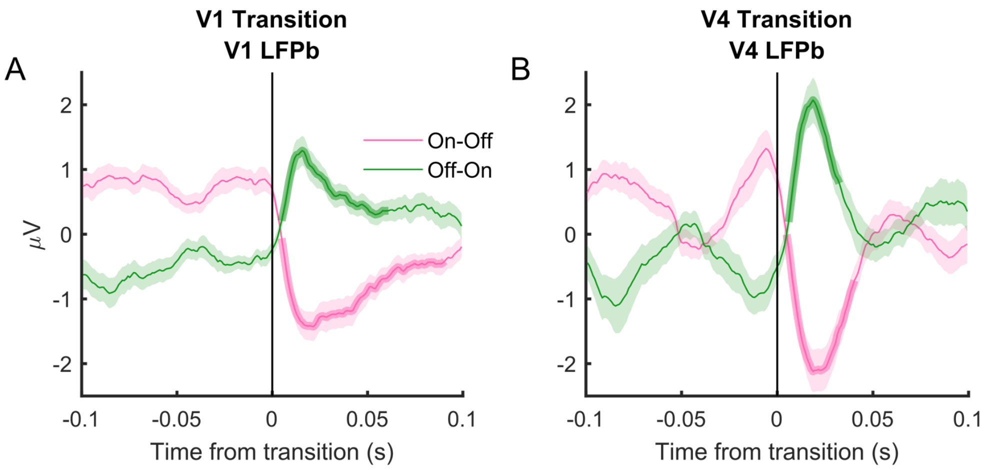

Figure S2. Relationship between local field potentials (LFPs) and On-Off transitions, related to STAR Methods. Bipolar re-referenced LFP activity aligned to state transitions for V1 (A) and V4 (B), averaged across channels and recordings. Only epochs without transitions preceding or following the alignment transition within 100 ms were included. Thick green and pink lines indicate the times the activity was higher (green) or lower (pink) than the average activity (FDR-corrected, one-sided Wilcoxon signed rank test). Data are represented as mean ± SEM across recordings.

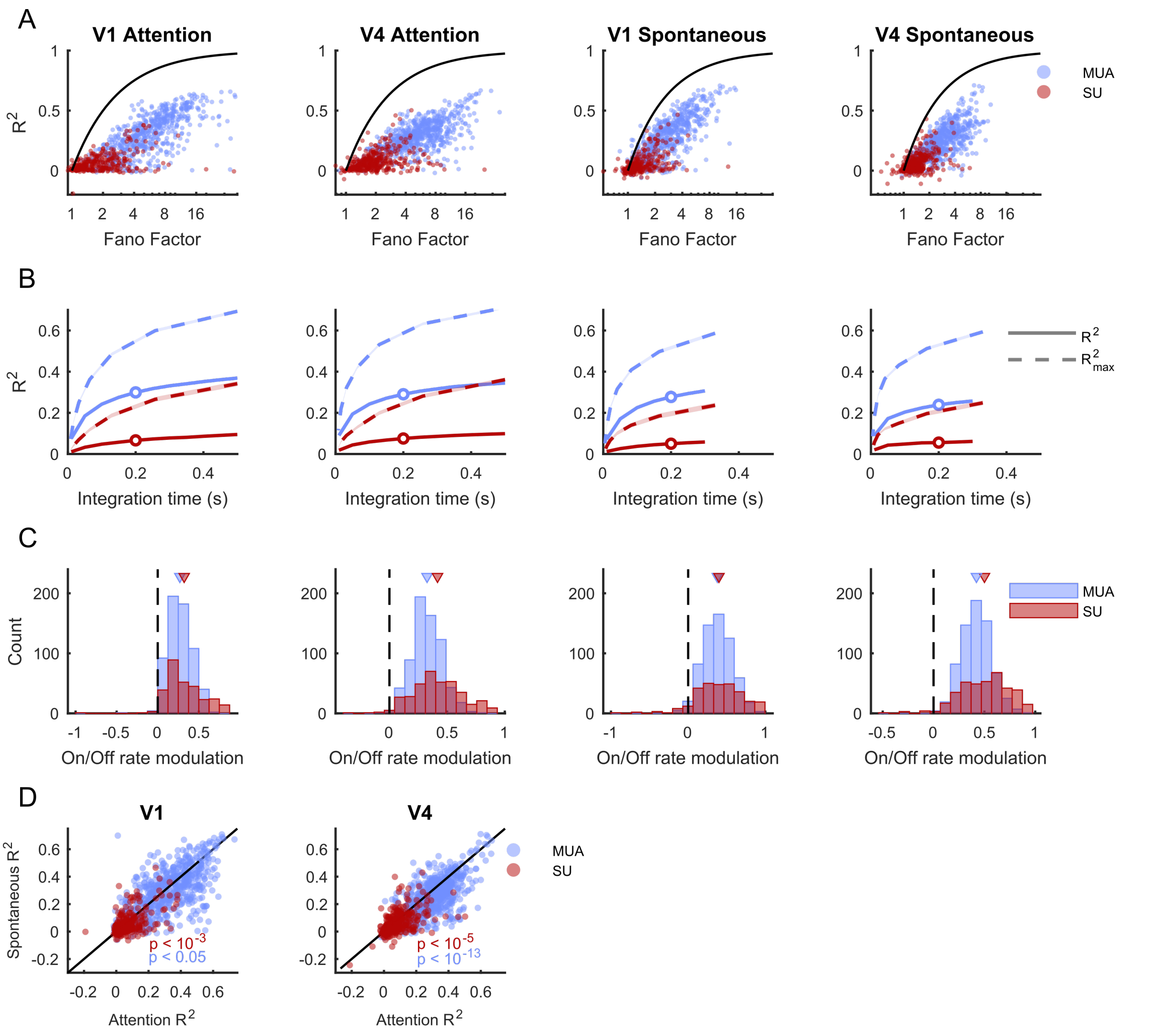

Figure S3. Variance explained by On-Off transitions, related to STAR Methods. (A) Scatter plot of the variance explained by the HMM versus the spike-count variability measured by the Fano factor for single- (SU, red dots) and multi-units (MU, blue dots). Each dot represents the average across all trials of one recording. Solid black line depicts the maximal explainable variance for each Fano factor value. (B) Population-average of the variance explained by the HMM as a function of the integration time window for single- (SU, red line) and multi-units (MU, blue line). Dashed lines depict the corresponding maximal explainable variance. White circles indicate 200 ms integration time window that was used in panel A. Error bars are ±SEM. (C) Distribution of the On/Off firing rate modulation index ([ron − roff]/[ron + roff]) for single- (SU, red) and multi-units (MU, blue). From left to right is plotted V1 during attention, V4 during attention, V1 during fixation (spontaneous activity) and V4 during fixation. (D) Variance explained in spontaneous activity plotted against that during attention for V1 (left) and V4 (right). Statistics: two-sided Wilcoxon signed rank tests.

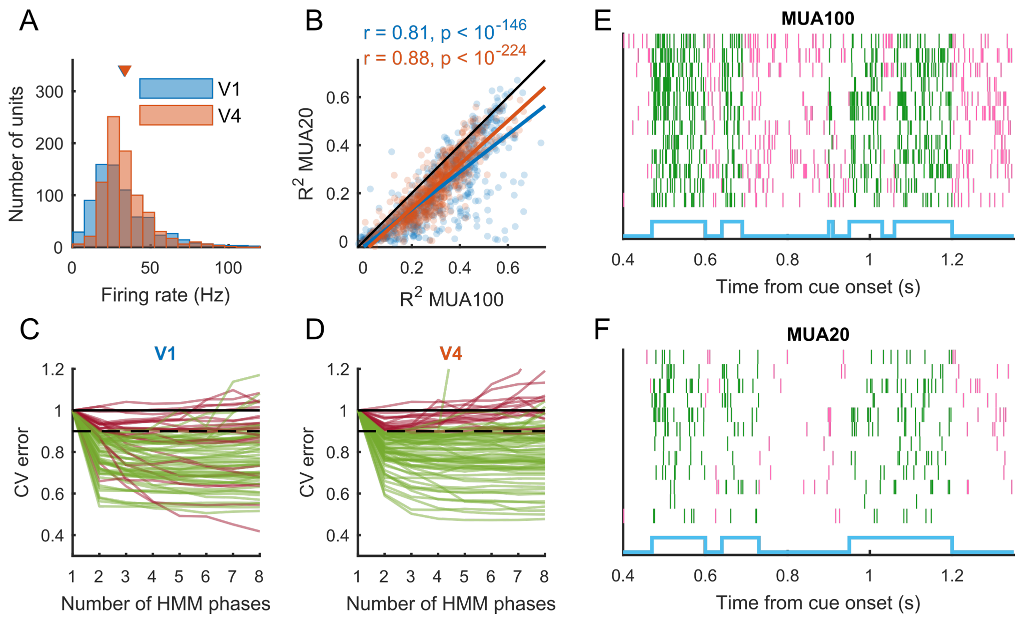

Figure S4. MUA definition control analysis, related to STAR Methods. (A) Trial-averaged firing rate in the period between 400 ms after cue onset until first dimming for MUA extracted based on a 20 Hz firing rate during the spontaneous period. (B) Variance explained (R^2) by the HMM for the original 100 Hz data (MUA100) plotted against R^2 for MUA extracted based on spontaneous activity (MUA20) for V1 (blue) and V4 (red). Each marker depicts one channel. The HMM fit is highly similar, as shown by their strong correlation indicated by the blue and red line. Statistics: Pearson correlation. As most markers fall below the black unity line, R^2 is higher for MUA100. (C-D) Cross validation (CV) error for MUA extracted based on spontaneous activity plotted against the number of phases in each HMM for V1 (C) and V4 (D). Compare to Figure S1A & C. (E-F) Example trial across-laminar raster plot and the corresponding latent state (blue) for MUA100 (E) and for MUA20 (F).

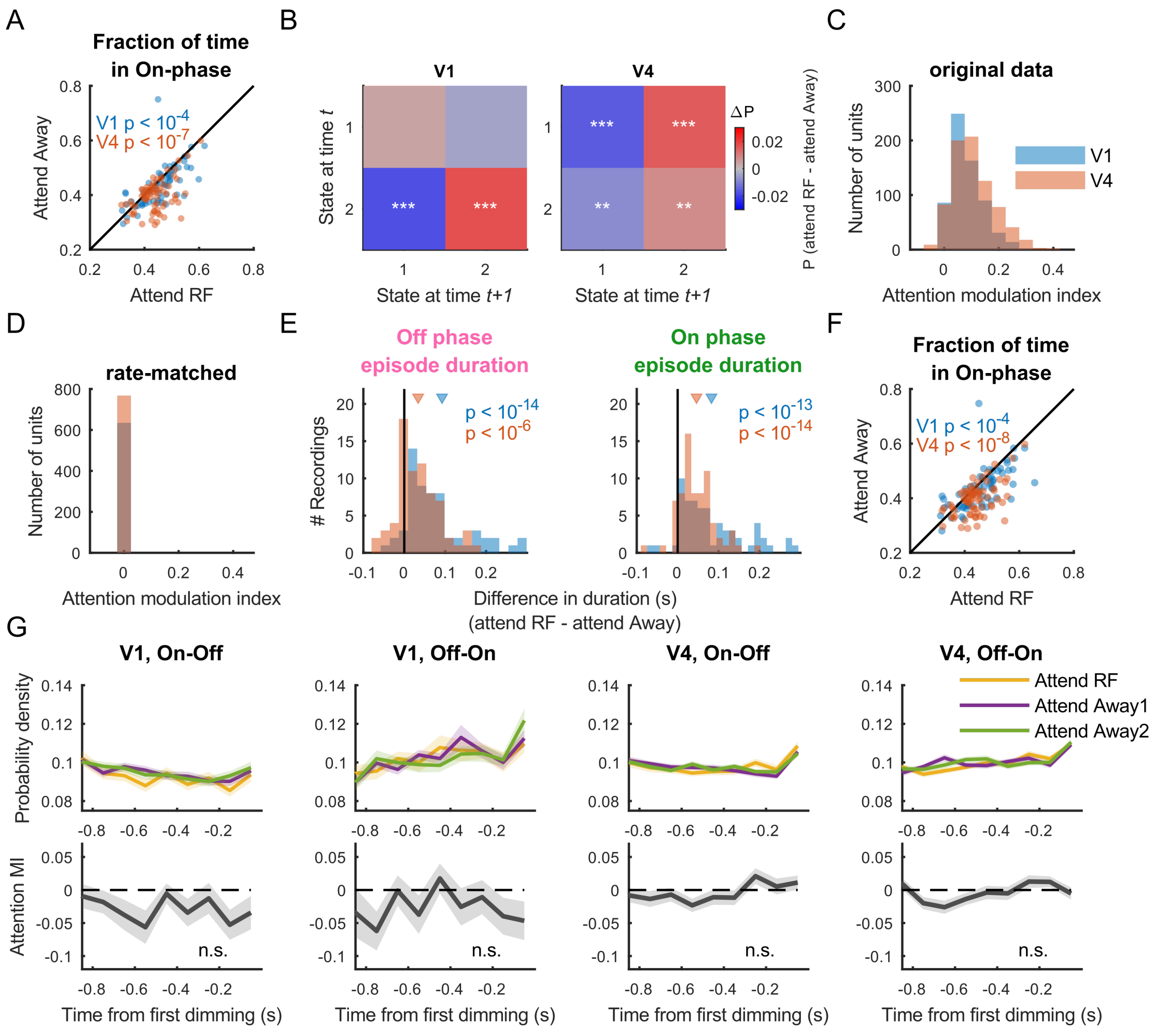

Figure S5. Attentional modulation of HMM parameters and rate-matching control, related to Figure 1 and STAR Methods. (A) The fraction of time spent in an On phase is increased when attention is directed towards the RFs. (B) Attentional influence on HMM transition probabilities. Shown is the difference between transition matrices (attend RF – attend Away). (C-F) Rate-matching control analysis. (C) Distribution of attention modulation index of the mean firing rate of multi-units for V1 (blue) and V4 (red). Trial-averaged mean firing rate was higher in attend RF conditions compared to attend away conditions. (D) As in C, but after rate-matching across attention conditions. (E) Distribution of the difference ($\tau_{Attend\ RF} - \tau_{Attend\ away}$) in average duration of the On (right panel) and Off (left panel) episodes between attend RF and attend away conditions after rate matching. The increase in On episode durations is preserved in the rate-matched data, thus this effect was not an artifact of higher mean firing rates during attention conditions. (F) The increased fraction of time spent in an On phase when attention is directed towards the RFs is preserved in the rate-matched data. (G) Transition probability density function (PDF) for phase transitions aligned to the first-dimming event (top) and its attentional modulation index

(MI, bottom; $MI = (Attend_{RF} - Attend_{Away})/(Attend_{RF} + Attend_{Away})$). From left to right: V1 On-Off transitions, V1 Off-On transitions, V4 On-Off transitions and V4 Off-On transitions. Data are represented as mean ± SEM across recordings. As no attention MI time bin deviated from zero, transition PDFs did not differ across attention conditions. Statistics: two-sided Wilcoxon signed rank tests; *, **, ***, indicate FDR corrected significance levels of $p < 0.05$, $p < 0.01$ and $p < 0.001$, respectively.

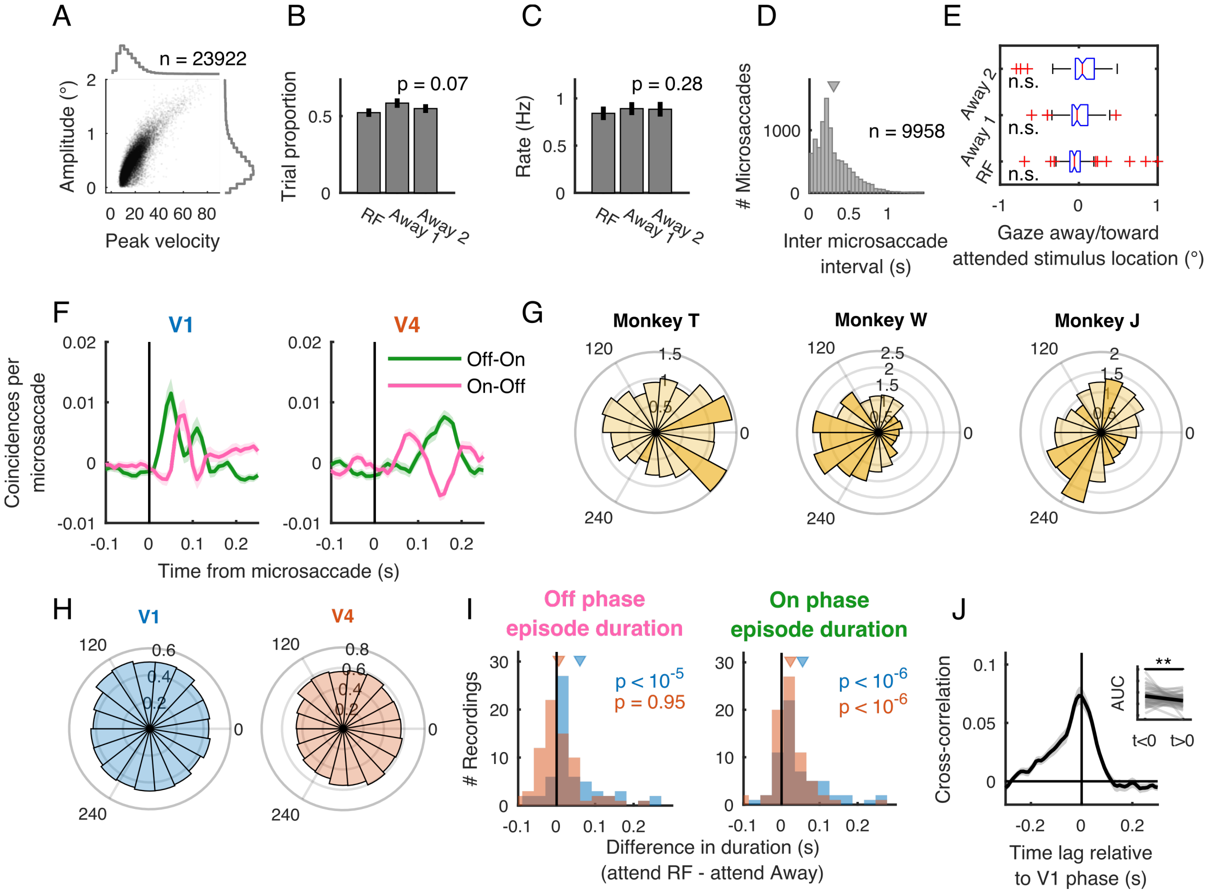

Figure S6. Relationship between microsaccades and On-Off transitions, related to Figure 1, Figure 2 and STAR Methods. (A) Microsaccade amplitude versus velocity plot. The histograms along the top (right) indicate the distribution of velocities (amplitudes). (B) Proportion of trials with microsaccades across attention conditions. (C) Microsaccade rate across attention conditions. (D) Distribution of inter-microsaccade-intervals. (E) Average gaze on trials without microsaccades relative to the location of the attended stimulus. Raw gaze locations were rotated such that the attended stimulus location was aligned to 0°, the x-direction therefore indicates gaze towards or away from the stimulus. (F) Cross-correlation of On-Off transitions in V1 (left) and V4 (right) triggered to microsaccade onset. (G) Relative microsaccade frequency between attention and control conditions across microsaccade directions, aligned to the RF location for each subject. The relative frequency is computed for each direction (in 20° bins) as the ratio of the number of microsaccades towards that direction in attention over control conditions. 0° corresponds to the RF location, which aligns with the attended stimulus in the attend RF condition. 120 and 240° correspond to the attend away stimulus locations. Directions with significantly higher relative microsaccade frequency are highlighted with darker color-fill (chi-squared residuals test at 0.05 significance level with Bonferroni correction). Microsaccades were not systematically directed towards or away from the RF location across subjects. (H) Fraction of microsaccades that were followed by Off-On transitions across

microsaccade directions, aligned to the RF location. The fraction of microsaccades followed by transitions did not significantly vary across microsaccade directions (chi-squared test, V1: $P=0.99$; V4: $P=0.38$). (I) The increase of On-episode durations in V1 (blue) and V4 (red), and the increase in Off-episode durations in V1 is preserved after exclusion of trials in which microsaccades occurred. (J) Cross-correlation between time series of On-Off dynamics in V1 and V4 after exclusion of trials in which microsaccades occurred. Statistics: two-sided Wilcoxon signed rank test (panel E, I and J) and a repeated measures ANOVA (panel B and C). Data are represented as mean ± SEM across recordings; *, ** and *** indicate significance levels of $p < 0.05$, $p < 0.01$ and $p < 0.001$ respectively.

	Red	Green	Blue
**Monkey 2 & 3, and monkey 1 (n=4)**	a. [220 0 0] – 12.8<br>b. [140 0 0] – 4.2	a. [0 135 0] – 12.9<br>b. [0 90 0] – 4.6	a. [60 60 255] – 12.2<br>b. [30 30 180] – 4.6
**Monkey 1 (n=1)**	b. [170 0 0] –6.7	b. [0 105 0] – 6.4	b. [37 37 210] – 6.6
**Monkey 1 (n=1)**	b. [175 0 0] –7.2	b. [0 105 0] – 6.4	b. [40 40 220] – 7.7
**Monkey 1 (n=8)**	b. [180 0 0] –7.7	b. [0 110 0] – 7.3	b. [40 40 220] – 7.7

Table S1. Color values used for the three colored gratings across recording sessions and subjects, related to STAR Methods. Color values are indicated as [RGB] – luminance (cd/m^2). a = Undimmed values, b = Dimmed values. For monkey 1, we used a variety of dimmed values across recordings.
