# Supplementary material for: Top-down coordination of local cortical state during selective attention
Source: Neuron. 2021 Mar 3;109(5):894–904.e8. doi: 10.1016/j.neuron.2020.12.013 (PMC7927916; doi:10.1016/j.neuron.2020.12.013)
Supplement: Document S2. Article plus supplemental information [file mmc2.pdf]

Neuron

Article

Top-down coordination of local cortical state during selective attention

Highlights

- Cortical excitability fluctuations are coordinated across visual areas
- During selective attention, these fluctuations are driven in a top-down manner
- The degree of coordination depends on shared spatial representation
- Increased cortical state coordination predicts better behavioral performance

Authors

Jochem van Kempen,
Marc A. Gieselmann, Michael Boyd,
Nicholas A. Steinmetz, Tirin Moore,
Tatiana A. Engel, Alexander Thiele

Correspondence

jochem.van-kempen@newcastle.ac.uk

In Brief

How do fluctuations in ongoing brain activity interact with cognitive functions? In this study, van Kempen et al. show that fluctuations in neural excitability are coordinated between visual areas with retinotopic precision. Top-down attention drives interareal coordination along the reverse cortical hierarchy, predicting better behavioral performance with increased coordination.

van Kempen et al., 2021, Neuron *109*, 894–904
March 3, 2021 © 2020 The Author(s). Published by Elsevier Inc.
https://doi.org/10.1016/j.neuron.2020.12.013

CellPress

CellPress OPEN ACCESS

Neuron

Article

Top-down coordination of local cortical state during selective attention

Jochem van Kempen,[1,5,*] Marc A. Gieselmann,[1] Michael Boyd,[1] Nicholas A. Steinmetz,[2] Tirin Moore,[3] Tatiana A. Engel,[4] and Alexander Thiele[1]

[1]Biosciences Institute, Newcastle University, Newcastle upon Tyne NE1 7RU, UK
[2]Department of Biological Structure, University of Washington, Seattle, WA 98195, USA
[3]Howard Hughes Medical Institute, Stanford University, Stanford, CA 94305, USA
[4]Cold Spring Harbor Laboratory, Cold Spring Harbor, NY 11724, USA
[5]Lead contact
*Correspondence: jochem.van-kempen@newcastle.ac.uk
https://doi.org/10.1016/j.neuron.2020.12.013

SUMMARY

Spontaneous fluctuations in cortical excitability influence sensory processing and behavior. These fluctuations, long thought to reflect global changes in cortical state, were recently found to be modulated locally within a retinotopic map during spatially selective attention. We report that periods of vigorous (On) and faint (Off) spiking activity, the signature of cortical state fluctuations, are coordinated across brain areas with retinotopic precision. Top-down attention enhanced interareal local state coordination, traversing along the reverse cortical hierarchy. The extent of local state coordination between areas was predictive of behavioral performance. Our results show that cortical state dynamics are shared across brain regions, modulated by cognitive demands and relevant for behavior.

INTRODUCTION

Cortical activity is not solely determined by external inputs but reflects ongoing fluctuations in neural excitability referred to as cortical state (Harris and Thiele, 2011; Kohn et al., 2009). Endogenous variability in cortical state shapes sensory responses and influences behavioral performance (Arieli et al., 1996; Gutnisky et al., 2017; McGinley et al., 2015a; Renart and Machens, 2014; Schölvinck et al., 2015). Although these fluctuations were long thought to be a global phenomenon that influences activity throughout the cortex (Harris and Thiele, 2011; Lee and Dan, 2012), recent evidence has revealed that signatures of cortical state are modulated locally within the retinotopic map in macaque mid-level visuo-cortical area V4 during selective attention (Engel et al., 2016).

Cortical state fluctuations manifest in periods of vigorous (On) and faint (Off) spiking activity occurring synchronously across cortical laminae. Spatially selective attention directed toward the receptive fields (RFs) of the neural population modulates On-Off dynamics by increasing the duration of On episodes (Engel et al., 2016). Thus, cognitive demands that selectively affect targeted retinotopic locations can modulate local signatures of global cortical state fluctuations. However, perception and cognition depend on the activity of many areas spanning the cortical hierarchy, which begs the question of whether cortical-state dynamics are coordinated across different brain regions during attention, whether this coordination progresses in a top-down or bottom-up manner, and whether it is relevant for behavior.

To investigate this, we recorded simultaneous activity from multiple brain regions spanning the cortical hierarchy. We discovered that the coordination of cortical state is retinotopically precise and progresses in a reverse hierarchical manner during selective attention. Enhanced interareal coordination of cortical state was moreover predictive of improved behavioral performance. These results show that temporally and spatially precise fluctuations in excitability are coordinated across networks of distributed areas across the cortical mantle and modulated by cognitive demands to serve behavioral goals.

RESULTS

We recorded simultaneously from V1 and V4 using 16-contact laminar electrodes while 3 rhesus macaques performed a feature-based spatial attention task (Figure 1A). Electrodes were inserted perpendicular to the cortical surface on a daily basis such that the RFs overlapped both across all channels within each area and between the two areas (Figures 1B and 1C). We characterized On-Off dynamics in each area individually by fitting a Hidden Markov Model (HMM) to the spike counts (10-ms bins) of multiunit activity (MUA) across included channels (Figure 1D; STAR methods). In line with previous reports for V4 (Engel et al., 2016), we found that a 2-phase model was the most parsimonious model for the majority of recordings (V1: 63 of 76 recordings [82.9%], V4: 72 of 78 recordings [92.3%], V1 and V4: 55 of 71 recordings [77.5%]; Figures S1A–S1D).

During these recordings, On-Off dynamics explained on average approximately half of the maximal explainable variance

 © 2020 The Author(s). Published by Elsevier Inc.
This is an open access article under the CC BY license (http://creativecommons.org/licenses/by/4.0/).

CellPress
OPEN ACCESS

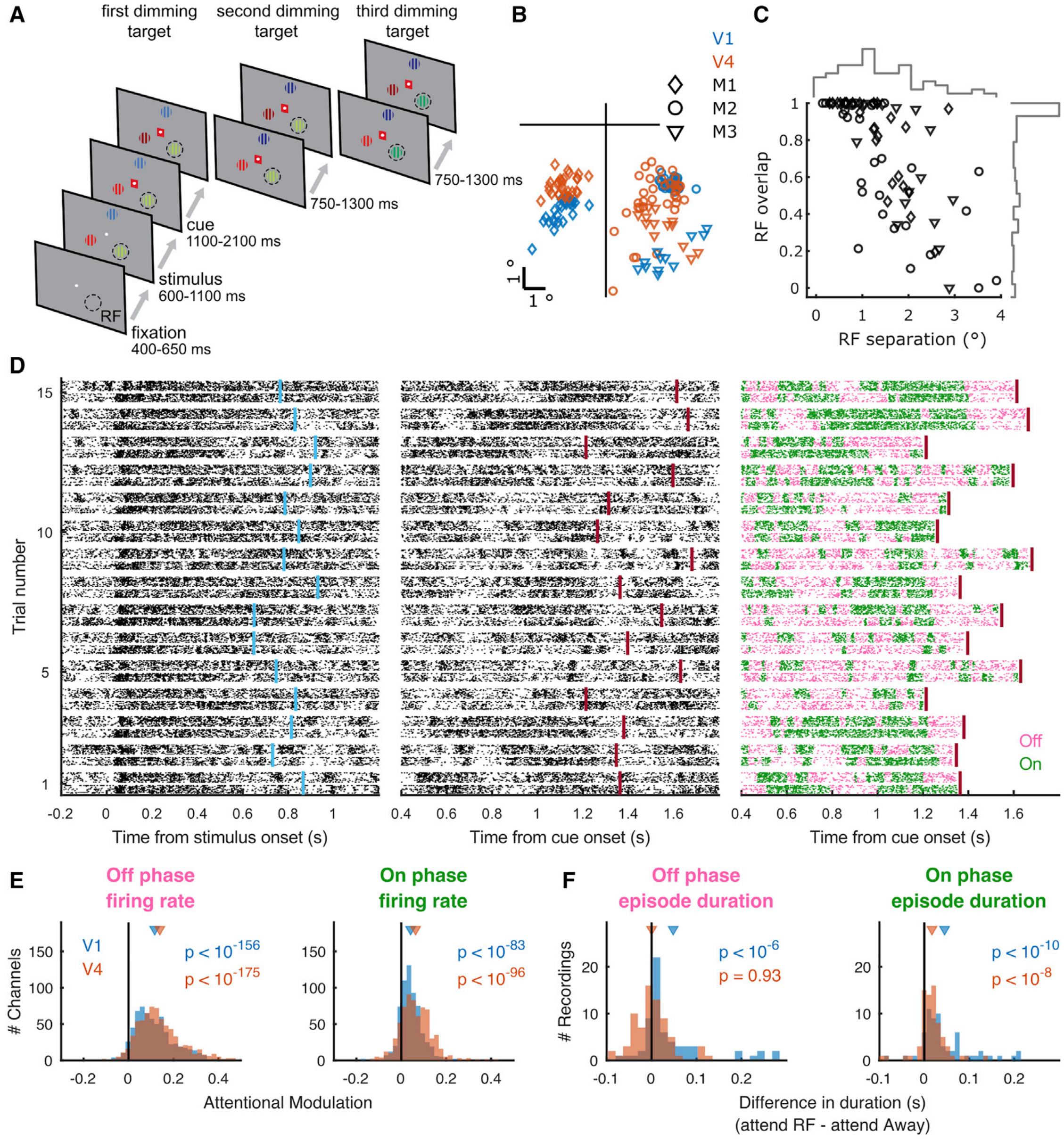

Figure 1. On-Off dynamics in V1 and V4 are modulated during selective attention

(A) Behavioral paradigm. The monkey held a lever to initiate the trial. Thereafter a central fixation spot was turned on. Upon fixation, 3 colored gratings appeared; one was presented inside the receptive fields (RFs) of the V1 neurons. After a variable delay, a cue matching one of the grating colors surrounded the fixation spot, indicating which grating was behaviorally relevant (target). In pseudorandom order, the stimuli decreased in luminance (dimmed). Upon dimming of the target, the monkey had to release the lever.

(B) Average RF center locations (across channels) for each recording, separately for each subject (M1–M3) and area.

(C) RF separation between V1 and V4 plotted against their overlap, expressed as the proportion of the V1 RF. The histograms along the top (right) indicate the distribution of RF separation (overlap) across all of the recordings.

(D) Raster plot of HMM fit to population activity (MUA) in V1 and V4. Simultaneously recorded multiunit spiking activity on 16-contact laminar electrodes in V1 and V4 for 15 example trials, aligned to stimulus (left) and cue onset (center and right). Each trial shows across laminar activity in V1 (bottom) and V4 (top), as raster plots (left 2 columns) color coded according to HMM estimation of On and Off phases (right). Center and right columns depict the same activity. The HMM was fit from 400 ms after cue onset to 30 ms after the first dimming event. Cue onset and first dimming are indicated for each trial by blue and red vertical bars, respectively.

(E) Attention increases firing rates during Off and On phases, both in V1 and V4.

(F) Attention increases the duration of On episodes, both in V1 and V4, whereas it increases the duration of Off episodes only in V1.

Statistics: two-sided Wilcoxon signed rank test.

See also Figures S1–S6.

(R^2_{max}; STAR methods) for both MUA as well as single-unit (SU) activity and both during fixation without visual stimulation as well as stimulus presentation, although R^2 was slightly higher during stimulus presentation (Figure S3). On-Off dynamics occurred without any obvious periodicity (Figure S1E), supporting the notion that On-Off dynamics are not the product of oscillatory activity but occur stochastically. In addition, On-Off transitions were phase locked to low-frequency fluctuations in local field potentials (LFPs), and the On-Off transitions in spike rate preceded changes in the LFP polarity (Figure S2). Furthermore, although the characterization of cortical-state dynamics with HMMs was better using population activity with high firing rates, it was not dependent on the specific MUA extraction parameters. We found highly similar results using a different MUA definition (20 Hz spontaneous activity) that resulted in much lower firing rates (Figure S4). Thus, in addition to V4 (Engel et al., 2016), On-Off dynamics also occur in V1, demonstrating that these dynamics are a general feature across multiple regions along the visuo-cortical hierarchy.

On-Off dynamics are modulated during selective attention

When attention was directed toward the RFs under study, firing rates were higher during both Off and On epochs in both areas (two-sided Wilcoxon signed rank test; V1: Off $p = 10^{-156}$, On $p = 10^{-83}$, V4: Off $p = 10^{-175}$, On $p = 10^{-96}$) (Figure 1E). In addition, On epoch durations increased in both V1 and V4 (two-sided Wilcoxon signed rank test; V1 $p = 10^{-10}$, V4 $p = 10^{-8}$) and Off epoch durations increased in V1 but not V4 (two-sided Wilcoxon signed rank test; V1: $p = 10^{-6}$, V4 $p = 0.93$) (Figure 1F). Critically, when attention was directed toward the RFs, more time was spent in an On phase (Figure S5A). Also, in line with the increased On epoch duration (and consistent with the HMM assumptions), transitions to an On phase were more likely during attention (Figure S5B). The attentional modulation of On-Off dynamics could not be explained by firing rate differences across attention conditions (Figures S5C–S5F) or microsaccades (Figure S6) and was furthermore independent of task timings (e.g., a non-flat hazard rate for stimulus dimming) as the probability of phase transitions over time did not systematically differ across attention conditions (Figure S5G). Thus, spatially selective attention modulated On-Off dynamics in a retinotopically precise manner in both V1 and V4 by increasing the duration of On epochs as well as the probability of being in an On phase.

Interareal coordination of On-Off dynamics

We next examined whether these spontaneous transitions were coordinated across visual areas. We computed cross-correlations between the V1 and V4 time series of On-Off phases (as estimated by the HMMs) during fixation (before stimulus and attention cue onset) and during directed attention (after cue onset, across attention conditions). During fixation, V1 and V4 transitions were coordinated but without either area leading/lagging behind the other (two-sided Wilcoxon signed rank test; $p = 0.12$) (Figure 2A). During attention, the coordination between V1 and V4 was enhanced, whereby On-Off transitions more often occurred in V4 first, before they were followed in V1, as evident from the skew toward negative values of the V4 relative to V1 transition times (two-sided Wilcoxon signed rank test; $p < 10^{-5}$) (Figure 2A). The cross-correlation strength and skew were independent of microsaccades (Figure S6). The strength was inversely related to both the overlap (Pearson correlation, $r = 0.28$, $p = 0.041$) and the separation between V1 and V4 RFs (Pearson correlation, $r = -0.36$, $p = 0.008$) (Figure 2B). Thus, the strength of On-Off dynamics coordination between visual areas is coupled to their retinotopic alignment.

To further characterize this interareal coordination, we computed average firing rates in V1 aligned to On-Off transition times in V4 and vice versa. In line with transitions being driven in a top-down manner, V1 firing rate changes followed V4 transitions whereas V4 firing rate changes preceded V1 transitions (Figure 2C). Differences in transition characteristics across V1 and V4 were not due to rate disparities across these areas, as neither single-unit firing rates (two-sided Wilcoxon rank-sum test, $p = 0.38$) nor variance explained by the HMM (two-sided Wilcoxon rank-sum test, $p = 0.25$) differed between V1 and V4.

We also analyzed spiking activity simultaneously recorded with 16-contact linear electrodes inserted perpendicular to layers in V4 and tangential to layers in the frontal eye field (FEF) (or with single electrodes in FEF in some sessions) from two monkeys performing a selective attention task (V4 data reported previously; Engel et al., 2016). This analysis revealed that changes in FEF firing rates precede On-Off transitions in V4 (Figure 2D). These results suggest that On-Off transitions traverse from higher to lower areas along the visual hierarchy during selective attention.

To investigate the relationship between V1 and V4 On-Off transitions more closely, we fit a 4-state HMM to V1 and V4 data simultaneously (HMM_{V1-V4}), with the 4 HMM states defined as (state 1) $V1_{off}$–$V4_{off}$, (state 2) $V1_{on}$–$V4_{off}$, (state 3) $V1_{off}$–$V4_{on}$ and (state 4) $V1_{on}$–$V4_{on}$ (Figure 3A). This model allowed us to investigate two specific scenarios (Figure 3B). In the first scenario (yellow), we asked: from a situation in which both areas are in an Off phase (state 1), is it more likely for V1 (state 2) or V4 (state 3) to transition to an On phase first? The second scenario (purple) addresses a related question: from a situation in which both areas are in an On phase (state 4), is it more likely for V1 (state 3) or V4 (state 2) to transition to an Off state first? The transition probabilities (Figures 3C and 3D) revealed that when both areas were in an Off phase, it was more likely for V4 to transition to an On phase first (two-sided Wilcoxon signed rank test; $p < 10^{-3}$). Likewise, if both areas were in an On phase, it was more likely for V4 to transition to an Off phase first (two-sided Wilcoxon signed rank test; $p < 10^{-3}$). Thus, when both areas are in the same phase, it is more likely for V4 to transition away from this phase first. This finding was, however, not specific to the attend RF condition, as we found similar results for each individual attention condition (attend RF and attend away), as well as during fixation (data not shown). Selective attention directed toward the RF, however, modulated the transition probabilities from the yellow scenario. Specifically, it decreased the probability of transitioning from state 1 to state 2, and increased the probability of transitioning from state 1 to state 3 (two-sided Wilcoxon signed rank test; $p < 10^{-3}$) (Figures 3E and 3F). Selective attention directed toward the RF thus specifically increases the retinotopically precise, top-down drive of

CellPress
OPEN ACCESS

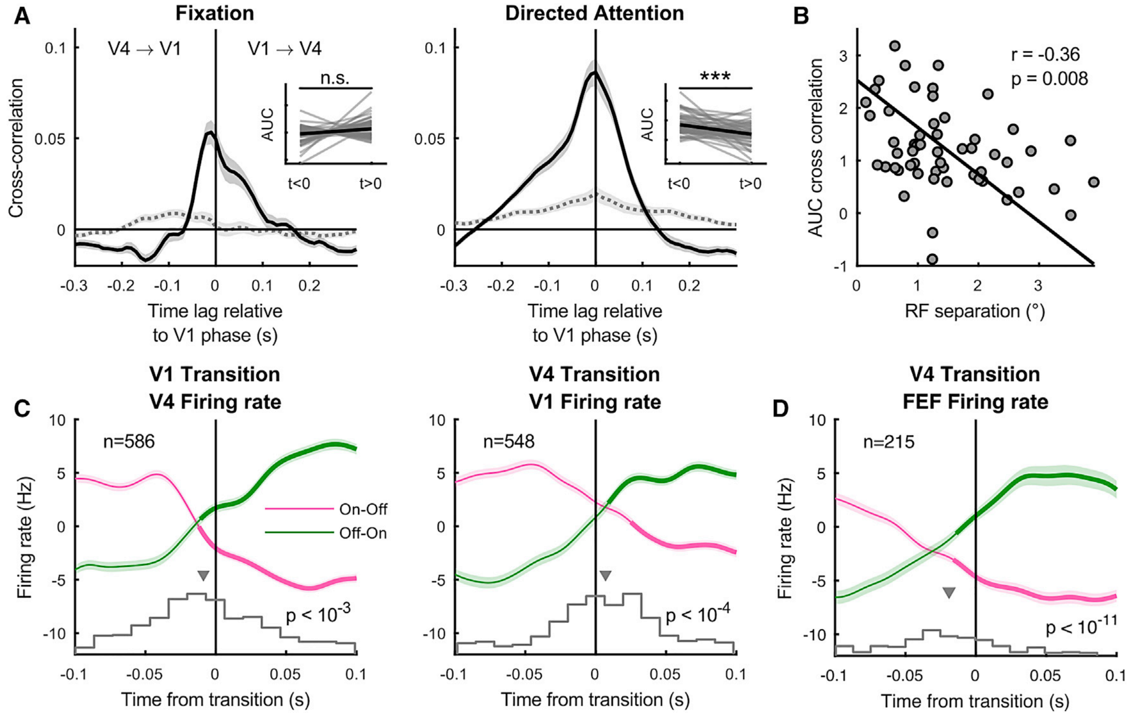

Figure 2. Interareal coordination of cortical state

(A) Cross-correlation between time series of On-Off phases in V1 and V4 relative to V1 phase during fixation (left) and after cue onset (right). Insets show the area under the cross-correlation curve for times smaller and larger than zero. The dashed gray line depicts the shuffle predictor.

(B) RF separation plotted against the area under the cross-correlation curve during attention (from the right panel of A). The line indicates the standardized major axis regression fit.

(C) Spiking activity in one area aligned to state transitions in the other area, averaged across channels and recordings. Only epochs without transitions preceding or following the alignment transition within 100 ms were included. Thick green and pink lines indicate the times the firing rate was higher (green) or lower (pink) than the average rate (false discovery rate [FDR] corrected, one-sided Wilcoxon signed rank test). Along the bottom are the histograms of the crossing point of 2 straight lines fit (least-squares) to the transition-aligned multiunit firing rate.

(D) Conventions as in (C), but from a different dataset in which activity was recorded simultaneously from V4 and FEF.

Statistics: two-sided Wilcoxon signed rank test (A, C, and D), Pearson correlation (B). Data are represented as means ± SEMs across channels.

See also Figure S6.

transitions from an Off to an On phase and coordinates these transitions between areas.

Finally, this model revealed that, although On-Off phases/transitions are correlated, each area spends a substantial fraction of time in opposite phases (Figure 3G). Selective attention decreased the time spent in state 1 whereas it increased the time spent in state 3 and state 4, i.e., the states where V4 was in an On phase (two-sided Wilcoxon signed rank test; state 1 $p < 10^{-4}$, state 2 $p = 0.61$, state 3 $p < 10^{-2}$, state 4 $p < 10^{-3}$) (Figure 3H). The increased On episode duration with attention toward the RF found for V1 (Figure 1F) was thus driven by the increased fraction of time spent in state 4, where V1 and V4 were coordinated, whereas the increased On episode duration within V4 was due to an increase in time spent across state 3 and state 4.

Spectral signatures of On-Off dynamics

We next investigated the relationship between On-Off dynamics and measures of cortical (de)synchronization using the bipolar re-referenced LFP. During On phases in either V1 or V4, low-frequency (<~20 Hz) LFP power was suppressed and high-frequency (>~20 Hz) power was increased, both in V1 and V4 (Figures 4A–4D). However, this desynchronization was not restricted to coordination within areas, but is sensitive to state transitions across areas. Specifically, LFP power spectra in both areas varied across the four states of HMM_{V1-V4} (Figures 4E and 4F). To demonstrate this, we investigated the difference in power spectra across states in which the On-Off phase within an area remained constant but differed in the other area. For example, we investigated the V1 LFP power spectra across states 1 and 3, wherein V1 was in an Off phase during both states, but V4 was either Off or On. This analysis revealed that the LFP power in V1 is modulated by V4 On-Off phase bidirectionally. If V1 was in either an On or an Off phase, a transition to an On phase in V4 increased V1 high-frequency power. A transition to an On phase in V1, however, only affected V4 high-frequency power when V4 was in an Off phase. When V4 was in an On phase, V1 phase did not affect high-frequency dynamics in V4. Thus, V4 phase influenced V1 LFP regardless of V1 phase, whereas V1 phase affected high-frequency dynamics in V4 only during

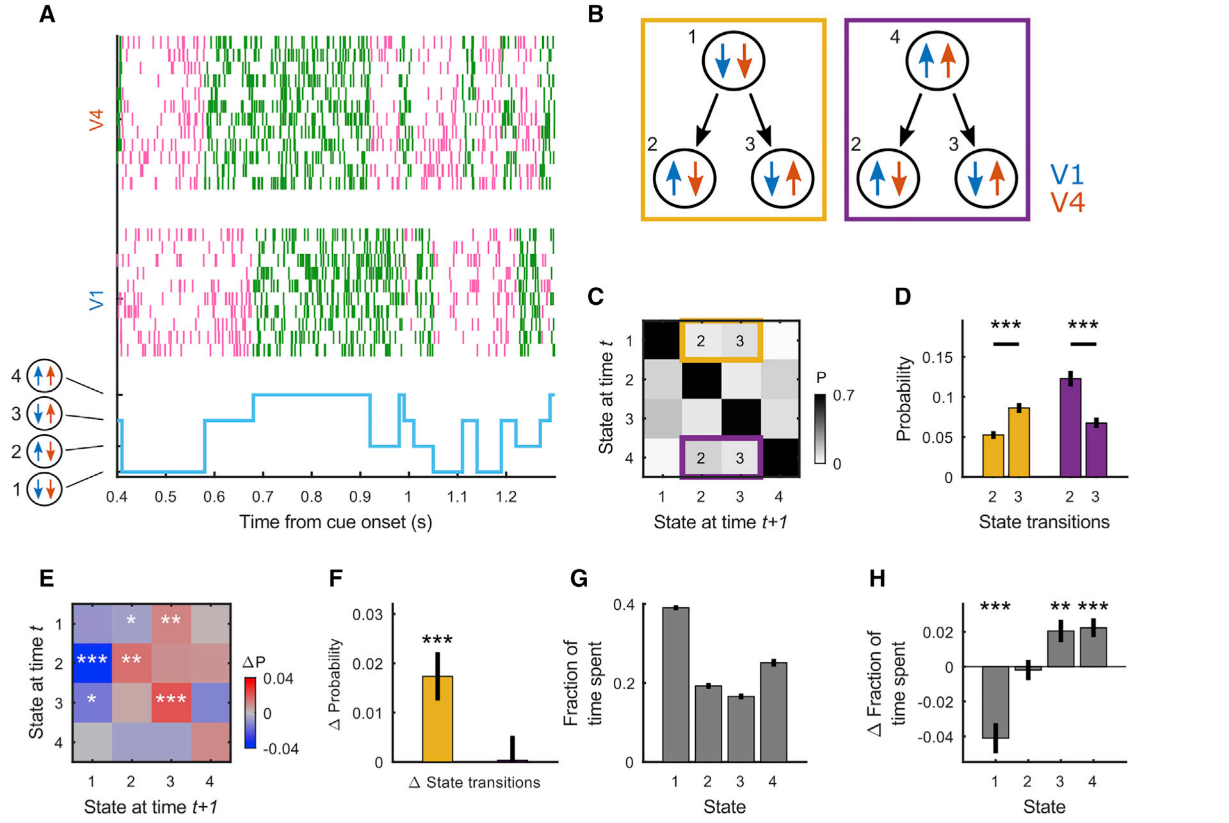

Figure 3. HMM with 4 states fit simultaneously to V1 and V4 data

(A) Example trial with the HMM state-trajectory (bottom) and across-laminar MUA raster plot for V1 (center) and V4 (top).

(B) Schematic describing scenarios for testing 2 questions: (1, left yellow box) from a state in which both V1 and V4 are Off, is it more likely for V1 or V4 to transition to the On phase first? (2, right purple box) From a state in which both V1 and V4 are On, is it more likely for V1 or V4 to transition to the Off phase first?

(C) HMM transition probability matrix, indicating the probability of staying in a state (diagonal) or transitioning from one state to another. Highlighted are scenarios set out in (B).

(D) Transition probabilities indicated in (B) and (C).

(E) Attentional influence on state-transition probabilities: the difference transition matrix (attend RF–attend away) is shown.

(F) Attentional influence (attend RF–attend away) on the difference between state transition probabilities (state 3–state 2) for each of the 2 scenarios indicated in (B)–(D). Selective attention increases the difference between the transition probabilities for states 2 and 3 for the yellow, but not the purple scenario.

(G) The fraction of time spent in each of the 4 states.

(H) The difference in time spent in each of the 4 states when attention is directed toward or away from the RF (attend RF–attend away).

Statistics: two-sided Wilcoxon signed rank test (FDR corrected); data are represented as means ± SEMs across recordings; significance levels *p < 0.05, **p < 0.01, and ***p < 0.001.

Off phases in V4. As firing rates of single units do not differ between V1 and V4 (two-sided Wilcoxon signed rank test; $p = 0.38$), these results cannot be explained by rate disparities across areas.

Thus, On phases are accompanied by a more desynchronized state, even if the area showing the desynchronization is itself not in an On (spiking) phase. Furthermore, in addition to state transitions and rate changes, as V1 LFP power is driven more by V4 state than vice versa, On-Off dynamics related high-frequency spectral changes also seem to be driven in a top-down manner during attention.

On-Off dynamics relate to global network state

In addition to selective attention, On-Off dynamics were linked to global arousal levels, as measured by pupil diameter (Aston-Jones and Cohen, 2005; McGinley et al., 2015a, 2015b; Reimer et al., 2014; Vinck et al., 2015). For each area individually, On epoch durations were longer on trials with larger baseline pupil diameter (Figures 5A–5C), in line with previous results (Engel et al., 2016). Furthermore, pupil diameter was predictive of On-Off dynamics coordination. Larger baseline pupil diameter was predictive of shorter epoch durations for $HMM_{V1–V4}$ state 1 (in which both areas were Off) and longer state 4 epoch durations (in which both areas were On) (Figure 5D). Central arousal, in addition to focused attention, thus specifically influenced epoch durations for states in which V1 and V4 phases were aligned. This is in line with pupil diameter being a proxy for central arousal, driving global network states that coordinate activity across distant brain areas. Importantly, as every trial is an attention trial, central arousal (unlike cortical state dynamics) should not differ across attention

CellPress
OPEN ACCESS

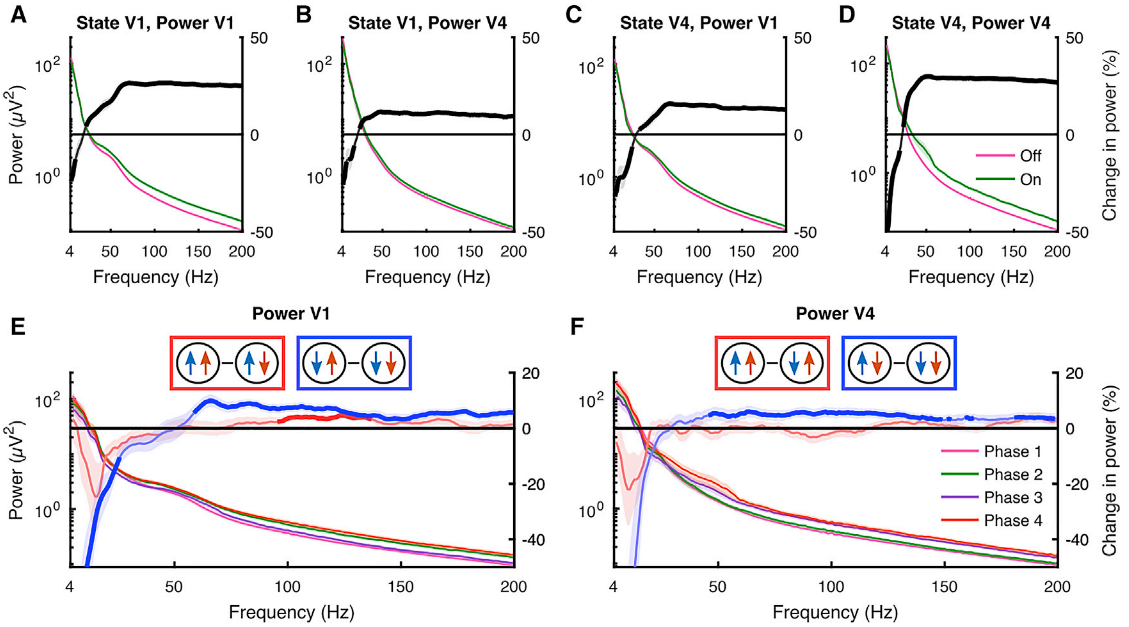

Figure 4. Bipolar re-referenced LFP power spectrum across HMM states
(A) Power in V1 during On and Off phases in V1.
(B) Power in V4 during On and Off phases in V1.
(C) Power in V1 during On and Off phases in V4.
(D) Power in V4 during On and Off phases in V4. Right y axis indicates the percentage of change in power during On versus Off phases (On-Off).
(E and F) Power spectrum in V1 (E) and V4 (F) for the 4-state HMM fit across V1 and V4 and the within-area power difference between On phases (red, V1: state 4-2; V4: state 4-3), or Off phases (blue, V1: state 3-1; V4: state 2-1). Only On/Off episodes of at least 250 ms were included. The thick percentage change lines indicate significantly modulated frequencies ($p < 0.05$, two-sided Wilcoxon signed rank test, FDR corrected).
Data are represented as means ± SEMs across recordings.

conditions. We verified this by showing that pupil diameter did not differ between attention conditions (Figure 5E). Furthermore, none of the effects of pupil diameter on epoch durations were dependent on RF separation (Pearson correlation [95% confidence interval, CI]; state 1: $-0.48 < r < 0.03$, p = 0.08; state 2: $-0.39 < r < 0.13$, p = 0.32; state 3: $-0.003 < r < 0.49$, p = 0.053; state 4: $-0.05 < r < 0.46$, p = 0.11). This shows that the effects of arousal and attention on On-Off dynamics are separable and independently controlled.

On phase coordination predicts better behavioral performance

We have demonstrated that the coordination of On-Off dynamics is retinotopically organized and driven in a top-down manner during selective attention. Is this organization also relevant for behavior? For both V1 and V4 individually, the On/Off phase at the time of target dimming was predictive of reaction time (RT) when the target was presented inside the RFs. We found an interaction between attention and On/Off phase (linear mixed effects model; V1 $\beta = 0.16 \pm 0.06$, p = 0.006; V4 $\beta = 0.12 \pm 0.06$, p = 0.045) with a main effect for phase (V1 $\beta = -0.27 \pm 0.09$, p = 0.002; V4 $\beta = -0.24 \pm 0.09$, p = 0.009), but no main effect of attention (V1 $\beta = -0.15 \pm 0.09$, p = 0.11; V4 $\beta = -0.06 \pm 0.09$, p = 0.48). Specifically, when either area was in an On phase when the target grating dimmed, RT was faster (two-sided Wilcoxon signed rank test; V1 p = 0.001, V4 $p < 10^{-3}$) (Figure 6A).

We furthermore found that On-Off phase coordination between V1 and V4, as assessed using HMM_{V1-V4}, was also predictive of behavioral performance. Again, we found an interaction between attention and On/Off phase (linear mixed effects model; $\beta = 0.07 \pm 0.02$, $p < 10^{-2}$), with a main effect of phase ($\beta = -0.14 \pm 0.04$, $p < 10^{-3}$) but not of attention ($\beta = -0.06 \pm 0.07$, p = 0.36). Performance was worst when at the time of target dimming both V1 and V4 were in an Off phase (state 1). Performance improved when either area was in an On phase, and it improved even further when both areas were in an On phase at the time of target dimming (Figure 6B). The coordination of On phases across visual areas is thus more beneficial for behavioral performance than the phase in either area alone.

DISCUSSION

We show that On-Off dynamics and their modulation by spatially selective attention are general features across multiple regions along the visuo-cortical hierarchy, occurring both in primary visual cortex (V1) and V4. The interareal coordination of On-Off dynamics occurs at a local retinotopic scale, which reflects the precision of anatomical connections, and is driven in a top-down manner across areas FEF, V4, and V1 during selective attention. Attention specifically facilitated top-down-driven transitions from Off to On states. Spectral analyses revealed that On-Off dynamics were associated with cortical (de)synchronization.

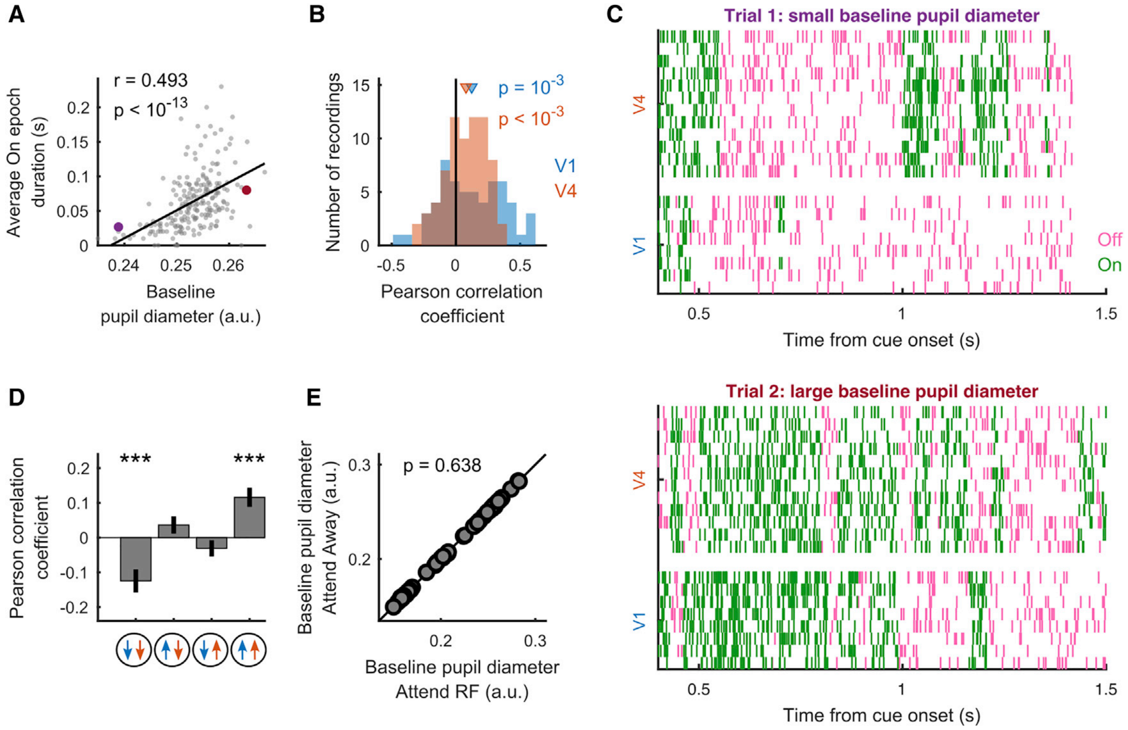

Figure 5. The relationship between baseline pupil diameter and On/Off episode durations

(A) Example recording showing that baseline pupil diameter is positively correlated to the average On episode duration in V1. Each dot represents a single trial; r is the Pearson correlation coefficient. The purple and red dots indicate the example trials used in (C).

(B) Across recordings, the average duration of On epochs in both V1 and V4 is positively correlated with the size of the baseline pupil diameter.

(C) MUA raster plot of 2 example trials in which the average On epoch duration is longer on the trial with larger (bottom) compared to the trial with smaller (top) baseline pupil diameter.

(D) Across recordings, baseline pupil diameter is negatively (positively) correlated with the average epoch duration when both V1 and V4 are in an Off (On) phase.

(E) The average baseline pupil diameter during attend RF conditions plotted against attend away conditions. There is no difference between attention conditions. Each dot represents a recording session.

Statistics: two-sided Wilcoxon signed rank test (FDR corrected) (B, D, and E) and Pearson correlation (A). Data are represented as means ± SEMs across recordings; significance levels *$p < 0.05$, **$p < 0.01$, and ***$p < 0.001$.

While central arousal also predicted the extent of interareal On-Off dynamics coordination, this occurred independently of attention-induced state coordination. Finally, we show that the coordination of On phases across visual areas is beneficial for behavioral performance.

On-Off dynamics rely on neuromodulatory drive and feedback projections

What drives the state coordination? Fluctuations in cortical state have previously been ascribed to neuromodulatory influences (Buzsaki et al., 1988; Constantinople and Bruno, 2011; Lee and Dan, 2012) and feedback projections (Rabinowitz et al., 2015; Zagha et al., 2013). The arousal linked state coordination in our data, inferred through pupil diameter analysis, is probably induced by changes in neuromodulatory tone (Aston-Jones and Cohen, 2005; Eldar et al., 2013; de Gee et al., 2017; Joshi et al., 2016; Murphy et al., 2014; Reimer et al., 2016; Varazzani et al., 2015). Changes in arousal level are linked to altered noradrenergic and cholinergic tone, which cause changes in cortical state (Harris and Thiele, 2011; Reimer et al., 2016). Direct stimulation (inhibition) of noradrenergic (Berridge and Foote, 1991; Berridge et al., 1993) or cholinergic nuclei (Metherate et al., 1992; Steriade et al., 1993) induce cortical depolarization (hyperpolarization) and desynchronization (synchronization). Stimulation-induced effects (as well as locomotion-induced effects) on cortical state are reversed by the local application of noradrenergic or cholinergic antagonists (Goard and Dan, 2009; Metherate et al., 1992; Pinto et al., 2013; Polack et al., 2013). Moreover, dopaminergic influences equally play a role, as ventral tegmental area (VTA) stimulation increased the duration of up states in the rat prefrontal cortex, an effect prevented by the systemic injection of a D1 receptor antagonist (Lewis and O'Donnell, 2000).

Neuromodulators are, however, not solely involved in changing levels of arousal, but are critically involved in attention-related signals in sensory and prefrontal areas (Dasilva et al., 2019; Herrero et al., 2008; Noudoost and Moore, 2011). The arousal- and attention-related actions of neuromodulators differentiate along tonic and phasic activities of the signaling neurons, whereby phasic signaling is associated with attention (Aston-Jones and Cohen, 2005; Parikh et al., 2007; Thiele and Bellgrove, 2018). Through this action, these neurons could in principle influence the attention-dependent coordination of the cortical state

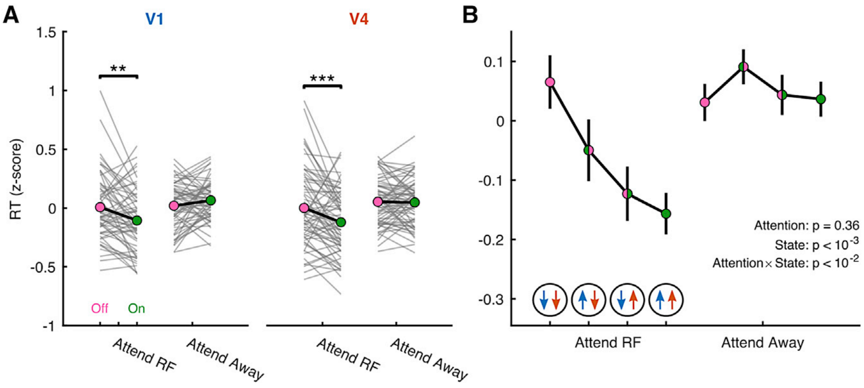

Figure 6. Across-area coordination of On-Off dynamics predicts behavioral performance

(A) On versus Off phase of population activity at the time of target dimming, determined individually for V1 and V4, predicts behavioral performance. RT decreases when attention is directed toward the RFs and either V1 or V4 is in an On phase.

(B) RT decreased from when both areas were Off, through V1 On–V4 Off, through V1 Off–V4 On, to V1 and V4 On when attention was directed toward the RFs.

Statistics: two-sided Wilcoxon signed rank test (A), and multilevel linear mixed-effects model (B). Data are represented as means ± SEMs across recordings; FDR corrected significance levels *p < 0.05,** p < 0.01, and ***p < 0.001.

that manifests in our data. However, the attention-induced coordination of the cortical state occurs in a reverse hierarchical manner, suggesting that it is mediated through feedback originating in the frontal cortex, not through global changes in neuromodulatory tone. It is thus more likely that attention-linked changes to neuromodulatory tone prepare areas to be more susceptible to top-down coordination of the cortical state. It may allow prefrontal circuits to be more effective in representing cognitive variables and affect sensory areas through feedback. For example, prefrontal acetylcholine and dopamine influence attentional signaling (Dasilva et al., 2019; Noudoost and Moore, 2011; Parikh et al., 2007). Adequate dopamine levels in FEF are critical to affect activity in V4 neurons in a similar manner to attention (Noudoost and Moore, 2011). Thus, neuromodulators interact with feedback signals, but the latter take a leading role in the fine tuning and task dependency of state coordination. The role of feedback in the coordination of synchronized versus desynchronized states has recently been shown through the activation and inactivation of vM1 in mice (Zagha et al., 2013). Critically, these feedback effects were pathway specific, they did not result in general (brain-wide) cortical state changes, they were rapid (~10 ms), and they were beneficial to sensory information coding in the affected sensory areas (Zagha et al., 2013). These results, in conjunction with ours, illustrate that feedback projections selectively influence the cortical state in sensory areas and that these network dynamics affect sensory stimulus processing.

Cognitive modulation of interareal activity coordination

The interareal coordination of On-Off dynamics and its relevance to behavioral performance suggests that trial-by-trial coordination of activity across brain regions is beneficial for information transfer and selectively modulated according to task demands. Stimulus-induced as well as spontaneously fluctuating oscillatory activities are correlated across areas according to both retinotopy and stimulus selectivity (Lewis et al., 2016). Neural excitability fluctuations thus follow the functional organization of the cortex. Selective attention modulates this interareal coherence (Bosman et al., 2012; Buschman and Miller, 2007; Gregoriou et al., 2009), potentially facilitating communication between hierarchically linked areas (Fries, 2005). Although attention can reduce within-area spike count correlations (Cohen and Maunsell, 2009; Herrero et al., 2013; Mitchell et al., 2009), depending on the signal correlation between neuronal pairs (Rabinowitz et al., 2015; Ruff and Cohen, 2014) and (in part) driven by On-Off dynamics (Engel et al., 2016; Shi et al., 2020), it increases correlated variability across functionally related areas (Oemisch et al., 2015; Ruff and Cohen, 2016). This increased coordination may be a prerequisite for successful interareal information transfer (Harris and Mrsic-Flogel, 2013) and may allow the propagation of sensory information to other brain regions (Luczak et al., 2013). It is tempting to speculate that this affects the organization of communication subspaces (Semedo et al., 2019), linking specific task-relevant neural ensembles in task-dependent manners.

Our results show that local cortical states are more synchronous across brain regions during attention (e.g., by better matched On epochs), and that attention directed toward the RF increases the top-down drive to transition to an On phase. When hierarchically linked areas are simultaneously active, potentially driven by the frontal cortex, global representation of information through recurrent processing could be facilitated, thereby aiding conscious stimulus processing (Baars, 2002; Dehaene and Changeux, 2011). The cognitive modulation of cortical state coordination could be a key component of this.

STAR★METHODS

Detailed methods are provided in the online version of this paper and include the following:

- KEY RESOURCES TABLE
- RESOURCE AVAILABILITY
  - Lead contact
  - Materials availability
  - Data and code availability
- EXPERIMENTAL MODEL AND SUBJECT DETAILS
  - Animals and procedures
  - Surgical preparation
- METHOD DETAILS
  - Behavioral paradigm

- Data acquisition and analysis
- Data preprocessing
- Receptive field estimation
- Bipolar re-referencing
- Attentional modulation
- Hidden Markov Model
- Variance explained by the HMM
- Dependence of On-Off dynamics on firing rates
- Dependence of On-Off dynamics on task timings
- Rate-matching control
- On-Off dynamics and behavioral performance
- Cross correlation
- Power estimation
- Microsaccades and their relation to On-Off dynamics

- QUANTIFICATION AND STATISTICAL ANALYSIS

SUPPLEMENTAL INFORMATION

Supplemental Information can be found online at https://doi.org/10.1016/j.neuron.2020.12.013.

ACKNOWLEDGMENTS

We thank Demetrio Ferro for advice on data analysis. Funded by the Wellcome Trust [093104] (J.v.K., M.A.G., and A.T.), MRC [MR/P013031/1] (J.v.K., M.A.G., and A.T.), NIH grants [R01 EB026949] (T.A.E.) and [EY014924] (N.A.S. and T.M.), and the Pershing Square Foundation (T.A.E.). This research made use of the Rocket High Performance Computing service at Newcastle University.

AUTHOR CONTRIBUTIONS

Conceptualization, J.v.K., T.A.E., and A.T.; Methodology, J.v.K. and T.A.E.; Formal Analysis, J.v.K.; Investigation, J.v.K., M.B., N.A.S., and A.T.; Data Curation, J.v.K.; Software, J.v.K., M.A.G., and T.A.E.; Writing – Original Draft, J.v.K.; Writing – Review & Editing, J.v.K., M.A.G., N.A.S., T.M., T.A.E., and A.T.; Resources, T.M. and A.T.; Supervision, M.A.G., T.A.E., and A.T.; Funding Acquisition, T.M. and A.T.; Visualization, J.v.K.

DECLARATION OF INTERESTS

The authors declare no competing interests.

Received: May 13, 2020
Revised: November 20, 2020
Accepted: December 16, 2020
Published: January 5, 2021

REFERENCES

Arieli, A., Sterkin, A., Grinvald, A., and Aertsen, A. (1996). Dynamics of ongoing activity: explanation of the large variability in evoked cortical responses. Science *273*, 1868–1871.

Aston-Jones, G., and Cohen, J.D. (2005). An integrative theory of locus coeruleus-norepinephrine function: adaptive gain and optimal performance. Annu. Rev. Neurosci. *28*, 403–450.

Baars, B.J. (2002). The conscious access hypothesis: origins and recent evidence. Trends Cogn. Sci. *6*, 47–52.

Bair, W., and O'Keefe, L.P. (1998). The influence of fixational eye movements on the response of neurons in area MT of the macaque. Vis. Neurosci. *15*, 779–786.

Benjamini, Y., and Yekutieli, D. (2001). The control of the false discovery rate in multiple testing under dependency. Ann. Stat. *29*, 1165–1188.

Berridge, C.W., and Foote, S.L. (1991). Effects of locus coeruleus activation on electroencephalographic activity in neocortex and hippocampus. J. Neurosci. *11*, 3135–3145.

Berridge, C.W., Page, M.E., Valentino, R.J., and Foote, S.L. (1993). Effects of locus coeruleus inactivation on electroencephalographic activity in neocortex and hippocampus. Neuroscience *55*, 381–393.

Bishop, C.M. (2006). Pattern Recognition and Machine Learning (Springer).

Bokil, H., Andrews, P., Kulkarni, J.E., Mehta, S., and Mitra, P.P. (2010). Chronux: a platform for analyzing neural signals. J. Neurosci. Methods *192*, 146–151.

Bosman, C.A., Schoffelen, J.-M., Brunet, N., Oostenveld, R., Bastos, A.M., Womelsdorf, T., Rubehn, B., Stieglitz, T., De Weerd, P., and Fries, P. (2012). Attentional stimulus selection through selective synchronization between monkey visual areas. Neuron *75*, 875–888.

Buschman, T.J., and Miller, E.K. (2007). Top-down versus bottom-up control of attention in the prefrontal and posterior parietal cortices. Science *315*, 1860–1862.

Buzsaki, G., Bickford, R.G., Ponomareff, G., Thal, L.J., Mandel, R., and Gage, F.H. (1988). Nucleus basalis and thalamic control of neocortical activity in the freely moving rat. J. Neurosci. *8*, 4007–4026.

Cohen, M.R., and Maunsell, J.H.R. (2009). Attention improves performance primarily by reducing interneuronal correlations. Nat. Neurosci. *12*, 1594–1600.

Constantinople, C.M., and Bruno, R.M. (2011). Effects and mechanisms of wakefulness on local cortical networks. Neuron *69*, 1061–1068.

Dasilva, M., Brandt, C., Gotthardt, S., Gieselmann, M.A., Distler, C., and Thiele, A. (2019). Cell class-specific modulation of attentional signals by acetylcholine in macaque frontal eye field. Proc. Natl. Acad. Sci. USA *116*, 20180–20189.

de Gee, J.W., Colizoli, O., Kloosterman, N.A., Knapen, T., Nieuwenhuis, S., and Donner, T.H. (2017). Dynamic modulation of decision biases by brainstem arousal systems. eLife *6*, e23232.

Dehaene, S., and Changeux, J.-P. (2011). Experimental and theoretical approaches to conscious processing. Neuron *70*, 200–227.

Eldar, E., Cohen, J.D., and Niv, Y. (2013). The effects of neural gain on attention and learning. Nat. Neurosci. *16*, 1146–1153.

Engbert, R., and Kliegl, R. (2003). Microsaccades uncover the orientation of covert attention. Vision Res. *43*, 1035–1045.

Engel, T.A., Steinmetz, N.A., Gieselmann, M.A., Thiele, A., Moore, T., and Boahen, K. (2016). Selective modulation of cortical state during spatial attention. Science *354*, 1140–1144.

Fries, P. (2005). A mechanism for cognitive dynamics: neuronal communication through neuronal coherence. Trends Cogn. Sci. *9*, 474–480.

Gieselmann, M.A., and Thiele, A. (2008). Comparison of spatial integration and surround suppression characteristics in spiking activity and the local field potential in macaque V1. Eur. J. Neurosci. *28*, 447–459.

Goard, M., and Dan, Y. (2009). Basal forebrain activation enhances cortical coding of natural scenes. Nat. Neurosci. *12*, 1444–1449.

Goettker, A., Braun, D.I., Schütz, A.C., and Gegenfurtner, K.R. (2018). Execution of saccadic eye movements affects speed perception. Proc. Natl. Acad. Sci. USA *115*, 2240–2245.

Gray, H., Bertrand, H., Mindus, C., Flecknell, P., Rowe, C., and Thiele, A. (2016). Physiological, Behavioral, and Scientific Impact of Different Fluid Control Protocols in the Rhesus Macaque (*Macaca mulatta*). eNeuro *3*, 1–15.

Gregoriou, G.G., Gotts, S.J., Zhou, H., and Desimone, R. (2009). High-frequency, long-range coupling between prefrontal and visual cortex during attention. Science *324*, 1207–1210.

Gutnisky, D.A., Beaman, C.B., Lew, S.E., and Dragoi, V. (2017). Spontaneous Fluctuations in Visual Cortical Responses Influence Population Coding Accuracy. Cereb. Cortex *27*, 1409–1427.

Harris, K.D., and Mrsic-Flogel, T.D. (2013). Cortical connectivity and sensory coding. Nature *503*, 51–58.

CellPress
OPEN ACCESS

Harris, K.D., and Thiele, A. (2011). Cortical state and attention. Nat. Rev. Neurosci. *12*, 509–523.

Herrero, J.L., Roberts, M.J., Delicato, L.S., Gieselmann, M.A., Dayan, P., and Thiele, A. (2008). Acetylcholine contributes through muscarinic receptors to attentional modulation in V1. Nature *454*, 1110–1114.

Herrero, J.L., Gieselmann, M.A., Sanayei, M., and Thiele, A. (2013). Attention-induced variance and noise correlation reduction in macaque V1 is mediated by NMDA receptors. Neuron *78*, 729–739.

Joshi, S., Li, Y., Kalwani, R.M., and Gold, J.I. (2016). Relationships between Pupil Diameter and Neuronal Activity in the Locus Coeruleus, Colliculi, and Cingulate Cortex. Neuron *89*, 221–234.

Jun, J.J., Steinmetz, N.A., Siegle, J.H., Denman, D.J., Bauza, M., Barbarits, B., Lee, A.K., Anastassiou, C.A., Andrei, A., Aydın, Ç., et al. (2017). Fully integrated silicon probes for high-density recording of neural activity. Nature *551*, 232–236.

Kohn, A., Zandvakili, A., and Smith, M.A. (2009). Correlations and brain states: from electrophysiology to functional imaging. Curr. Opin. Neurobiol. *19*, 434–438.

Lee, S.-H., and Dan, Y. (2012). Neuromodulation of brain states. Neuron *76*, 209–222.

Leopold, D.A., and Logothetis, N.K. (1998). Microsaccades differentially modulate neural activity in the striate and extrastriate visual cortex. Exp. Brain Res. *123*, 341–345.

Lewis, B.L., and O'Donnell, P. (2000). Ventral tegmental area afferents to the prefrontal cortex maintain membrane potential 'up' states in pyramidal neurons via D(1) dopamine receptors. Cereb. Cortex *10*, 1168–1175.

Lewis, C.M., Bosman, C.A., Womelsdorf, T., and Fries, P. (2016). Stimulus-induced visual cortical networks are recapitulated by spontaneous local and interareal synchronization. Proc. Natl. Acad. Sci. USA *113*, E606–E615.

Logothetis, N.K., Kayser, C., and Oeltermann, A. (2007). In vivo measurement of cortical impedance spectrum in monkeys: implications for signal propagation. Neuron *55*, 809–823.

Lowet, E., Gomes, B., Srinivasan, K., Zhou, H., Schafer, R.J., and Desimone, R. (2018). Enhanced Neural Processing by Covert Attention only during Microsaccades Directed toward the Attended Stimulus. Neuron *99*, 207–214.e3.

Luczak, A., Bartho, P., and Harris, K.D. (2013). Gating of sensory input by spontaneous cortical activity. J. Neurosci. *33*, 1684–1695.

Martinez-Conde, S., Macknik, S.L., Troncoso, X.G., and Hubel, D.H. (2009). Microsaccades: a neurophysiological analysis. Trends Neurosci. *32*, 463–475.

Martinez-Conde, S., Otero-Millan, J., and Macknik, S.L. (2013). The impact of microsaccades on vision: towards a unified theory of saccadic function. Nat. Rev. Neurosci. *14*, 83–96.

McGinley, M.J., David, S.V., and McCormick, D.A. (2015a). Cortical Membrane Potential Signature of Optimal States for Sensory Signal Detection. Neuron *87*, 179–192.

McGinley, M.J., Vinck, M., Reimer, J., Batista-Brito, R., Zagha, E., Cadwell, C.R., Tolias, A.S., Cardin, J.A., and McCormick, D.A. (2015b). Waking State: Rapid Variations Modulate Neural and Behavioral Responses. Neuron *87*, 1143–1161.

Metherate, R., Cox, C.L., and Ashe, J.H. (1992). Cellular bases of neocortical activation: modulation of neural oscillations by the nucleus basalis and endogenous acetylcholine. J. Neurosci. *12*, 4701–4711.

Mitchell, J.F., Sundberg, K.A., and Reynolds, J.H. (2009). Spatial attention decorrelates intrinsic activity fluctuations in macaque area V4. Neuron *63*, 879–888.

Mountcastle, V.B. (1957). Modality and topographic properties of single neurons of cat's somatic sensory cortex. J. Neurophysiol. *20*, 408–434.

Murphy, P.R., O'Connell, R.G., O'Sullivan, M., Robertson, I.H., and Balsters, J.H. (2014). Pupil diameter covaries with BOLD activity in human locus coeruleus. Hum. Brain Mapp. *35*, 4140–4154.

Noudoost, B., and Moore, T. (2011). Control of visual cortical signals by prefrontal dopamine. Nature *474*, 372–375.

Oemisch, M., Westendorff, S., Everling, S., and Womelsdorf, T. (2015). Interareal Spike-Train Correlations of Anterior Cingulate and Dorsal Prefrontal Cortex during Attention Shifts. J. Neurosci. *35*, 13076–13089.

Parikh, V., Kozak, R., Martinez, V., and Sarter, M. (2007). Prefrontal acetylcholine release controls cue detection on multiple timescales. Neuron *56*, 141–154.

Pettersen, K.H., Devor, A., Ulbert, I., Dale, A.M., and Einevoll, G.T. (2006). Current-source density estimation based on inversion of electrostatic forward solution: effects of finite extent of neuronal activity and conductivity discontinuities. J. Neurosci. Methods *154*, 116–133.

Pinto, L., Goard, M.J., Estandian, D., Xu, M., Kwan, A.C., Lee, S.-H., Harrison, T.C., Feng, G., and Dan, Y. (2013). Fast modulation of visual perception by basal forebrain cholinergic neurons. Nat. Neurosci. *16*, 1857–1863.

Polack, P.O., Friedman, J., and Golshani, P. (2013). Cellular mechanisms of brain state-dependent gain modulation in visual cortex. Nat. Neurosci. *16*, 1331–1339.

Rabiner, L.R. (1989). A tutorial on hidden Markov models and selected applications in speech recognition. Proc. IEEE *77*, 257–286.

Rabinowitz, N.C., Goris, R.L., Cohen, M., and Simoncelli, E.P. (2015). Attention stabilizes the shared gain of V4 populations. eLife *4*, e08998.

Reimer, J., Froudarakis, E., Cadwell, C.R., Yatsenko, D., Denfield, G.H., and Tolias, A.S. (2014). Pupil fluctuations track fast switching of cortical states during quiet wakefulness. Neuron *84*, 355–362.

Reimer, J., McGinley, M.J., Liu, Y., Rodenkirch, C., Wang, Q., McCormick, D.A., and Tolias, A.S. (2016). Pupil fluctuations track rapid changes in adrenergic and cholinergic activity in cortex. Nat. Commun. *7*, 13289.

Renart, A., and Machens, C.K. (2014). Variability in neural activity and behavior. Curr. Opin. Neurobiol. *25*, 211–220.

Roelfsema, P.R., Tolboom, M., and Khayat, P.S. (2007). Different processing phases for features, figures, and selective attention in the primary visual cortex. Neuron *56*, 785–792.

Ruff, D.A., and Cohen, M.R. (2014). Attention can either increase or decrease spike count correlations in visual cortex. Nat. Neurosci. *17*, 1591–1597.

Ruff, D.A., and Cohen, M.R. (2016). Attention Increases Spike Count Correlations between Visual Cortical Areas. J. Neurosci. *36*, 7523–7534.

Schölvinck, M.L., Saleem, A.B., Benucci, A., Harris, K.D., and Carandini, M. (2015). Cortical state determines global variability and correlations in visual cortex. J. Neurosci. *35*, 170–178.

Schroeder, C.E., Tenke, C.E., Givre, S.J., Arezzo, J.C., and Vaughan, H.G., Jr. (1991). Striate cortical contribution to the surface-recorded pattern-reversal VEP in the alert monkey. Vision Res. *31*, 1143–1157.

Schroeder, C.E., Mehta, A.D., and Givre, S.J. (1998). A spatiotemporal profile of visual system activation revealed by current source density analysis in the awake macaque. Cereb. Cortex *8*, 575–592.

Self, M.W., van Kerkoerle, T., Supèr, H., and Roelfsema, P.R. (2013). Distinct roles of the cortical layers of area V1 in figure-ground segregation. Curr. Biol. *23*, 2121–2129.

Semedo, J.D., Zandvakili, A., Machens, C.K., Yu, B.M., and Kohn, A. (2019). Cortical Areas Interact through a Communication Subspace. Neuron *102*, 249–259.e4.

Shi, Y.-L., Steinmetz, N.A., Moore, T., Boahen, K., and Engel, T.A. (2020). Influence of On-Off dynamics and selective attention on the spatial pattern of correlated variability in neocortex. BioRxiv. https://doi.org/10.1101/2020.09.02.279893.

Steriade, M., Amzica, F., and Nuñez, A. (1993). Cholinergic and noradrenergic modulation of the slow (approximately 0.3 Hz) oscillation in neocortical cells. J. Neurophysiol. *70*, 1385–1400.

Thiele, A., and Bellgrove, M.A. (2018). Neuromodulation of Attention. Neuron *97*, 769–785.

Thiele, A., Delicato, L.S., Roberts, M.J., and Gieselmann, M.A. (2006). A novel electrode-pipette design for simultaneous recording of extracellular spikes and iontophoretic drug application in awake behaving monkeys. J. Neurosci. Methods *158*, 207–211.

van Kempen, J., Boyd, M., and Thiele, A. (2020). Simultaneous laminar V1 and V4 recordings during selective attention. https://doi.gin.g-node.org/10.12751/g-node.b0mnn2/.

Varazzani, C., San-Galli, A., Gilardeau, S., and Bouret, S. (2015). Noradrenaline and dopamine neurons in the reward/effort trade-off: a direct electrophysiological comparison in behaving monkeys. J. Neurosci. *35*, 7866–7877.

Vinck, M., Batista-Brito, R., Knoblich, U., and Cardin, J.A. (2015). Arousal and locomotion make distinct contributions to cortical activity patterns and visual encoding. Neuron *86*, 740–754.

Zagha, E., Casale, A.E., Sachdev, R.N.S., McGinley, M.J., and McCormick, D.A. (2013). Motor cortex feedback influences sensory processing by modulating network state. Neuron *79*, 567–578.

CellPress
OPEN ACCESS

STAR★METHODS

KEY RESOURCES TABLE

REAGENT or RESOURCE	SOURCE	IDENTIFIER
Deposited data		
Electrophysiological data	Thiele lab	G-Node: https://doi.gin.g-node.org/10.12751/g-node.b0mnn2
Experimental models: organisms/strains		
Rhesus Macaque (*Macaca mulatta*)	Medical Research Council Centre for Macaques (MRC CFM)	N/A
Software and algorithms		
Analysis code	Thiele lab, Engel lab	https://gitlab.com/JvK/cortical-state-coordination
MATLAB	MathWorks	https://www.mathworks.com
Stimulus presentation and experimental control	NIH	Remote Cortex 5.95
Data acquisition	Neuralynx	Cheetah 5.6.3
Offline spike sorting	Neuralynx	SpikeSort3D
Other		
Laminar probe	Thiele lab: ATLAS neuro	E16R+R-150-S1-L10
Acquisition system	Neuralynx	Digital Lynx
Eye tracker	Arrington Research	Viewpoint MCU02

RESOURCE AVAILABILITY

Lead contact

Further information and requests for resources should be directed to and will be fulfilled by the Lead Contact, Jochem van Kempen (Jochem.van-Kempen@newcastle.ac.uk) .

Materials availability

This study did not generate new unique reagents.

Data and code availability

Preprocessed data necessary to replicate these results have been deposited to G-Node: https://doi.gin.g-node.org/10.12751/g-node.b0mnn2 (van Kempen et al., 2020). The MATLAB analysis code necessary to replicate these results is available at https://gitlab.com/JvK/cortical-state-coordination.

EXPERIMENTAL MODEL AND SUBJECT DETAILS

Animals and procedures

Subjects in our study were 3 male rhesus macaque monkeys (*Macaca mulatta,* age 10-12 years, weight 8.5-12.5 kg), housed under conditions described in detail previously (Gray et al., 2016). All animal procedures were performed in accordance with the European Communities Council Directive RL 2010/63/EC, the National Institute of Health's Guidelines for the Care and Use of Animals for Experimental Procedures, and the UK Animals Scientific Procedures Act. Animals were motivated to engage in the task through fluid control at levels that do not affect animal physiology and have minimal impact on psychological wellbeing (Gray et al., 2016).

Surgical preparation

The animals were implanted with a head post and recording chambers over area V1 and V4 under sterile conditions and general anesthesia. Surgical procedures and postoperative care conditions have been described in detail previously (Thiele et al., 2006).

METHOD DETAILS

Behavioral paradigm

Stimulus presentation and behavioral control was regulated by Remote Cortex 5.95 (Laboratory of Neuropsychology, National Institute for Mental Health, Bethesda, MD). Stimuli were presented on a cathode ray tube (CRT) monitor at 120 Hz, 1280 × 1024 pixels, at a distance of 54 cm. The location and size of receptive field (RF) were measured as described previously (Gieselmann and Thiele, 2008), using a reverse correlation method. Briefly, during fixation, a series of black squares (0.5-2° size, 100% contrast) were presented for 100 ms at pseudorandom locations on a 9 × 12 grid (5-25 repetitions for each location) on a bright background. RF eccentricity ranged from 3.4 to 7.5° in V1, and from 2.5 to 8.9° in V4.

During the main task (Figure 1A), the monkeys initiated a trial by holding a lever and fixating on a central white fixation spot (0.1°) displayed on a gray background (1.41 cd/m^2). After a fixed delay (614, 424, 674 ms, for monkeys 1, 2 and 3), three colored (for color values see Table S1) square wave gratings appeared equidistant from the fixation spot, one was centered on the RF of the V1 neurons under study. The locations of colored gratings were fixed for each recording session but were pseudorandomly assigned across sessions. Stimulus size varied between 2 and 4° diameter, depending on RF eccentricity and size. For most recordings we used drifting gratings but presented one monkey with stationary gratings during 22 out of 34 recording days. The drifting gratings moved perpendicular to the grating orientation, with the motion direction pseudorandomly assigned on every trial. After a random delay (618-1131 ms for monkey 1, 618-948 ms for monkeys 2 and 3; uniformly distributed), a central cue appeared that matched the color of one of the gratings, indicating that this grating would be behaviorally relevant on the current trial. After a variable delay (1162-2133 ms for monkey 1, 1162-1822 ms for monkeys 2 and 3; uniformly distributed), one of the pseudorandomly selected gratings changed luminance (for color values see Table S1), referred to as dimming. If the cued grating (target) dimmed, the monkey had to release the lever in order to obtain a reward. If, however, a non-cued grating (distractor) dimmed, the monkey had to ignore this and keep hold of the lever until the target dimmed on the second or third dimming event (each after another 792-1331 ms for monkey 1; 792-1164 ms for monkeys 2 and 3; uniformly distributed).

Data acquisition and analysis

We recorded from all cortical layers of visual areas V1 and V4 using 16-contact laminar electrodes (150 μm contact spacing, Atlas silicon probes). Out of a total of 76 V1 and 78 V4 recording sessions, 71 recordings were conducted simultaneously in both areas. The electrodes were inserted perpendicular to the cortex on a daily basis.

Raw data were collected using Remote Cortex 5.95 and by Cheetah data acquisition interlinked with Remote Cortex 5.95. Neuronal data were acquired with Neuralynx preamplifiers and a Neuralynx Digital Lynx amplifier. Unfiltered data were sampled with 24 bit at 32.7 kHz and stored to disc. Data were replayed offline, sampled with 16-bit and band-pass filtered at 0.5-300 Hz and down sampled to 1 kHz for local field potential (LFP) data, and filtered at 0.6-9 kHz for spike extraction. Eye position and pupil diameter was recorded at 220 Hz (ViewPoint, Arrington Research). Pupil diameter was recorded for 75 (90.4%) of recordings.

Simultaneous recordings from V4 and FEF were conducted using 16-channel U-Probes (Plexon) in both areas, or U-Probes in V4 and single electrodes in FEF. Probes were inserted perpendicular to layers in V4 and tangential to layers in the frontal eye field (FEF) from two monkeys performing a selective attention task. Details on data acquisition and processing have been described in detail previously (Engel et al., 2016). All data analyses were performed using custom written MATLAB (the Mathworks) scripts.

Data preprocessing

We corrected for any noise common to all channels via common average reference, in which the average of all channels is subtracted from each individual channel. We extracted population activity by progressively lowering spike extraction thresholds until approximately 100 Hz spiking activity was detected on each channel between fixation onset and the first dimming event. Well-isolated single units were obtained through manual spike sorting using SpikeSort3D (Neuralynx).

In order to determine signal-to-noise (SNR) ratios, recording stability and the visual response latency as well as for the computation of receptive fields (i.e., for preprocessing purposes only), we computed the envelope of MUA (MUAe) by low-pass filtering (< 300 Hz, fifth order Butterworth) the rectified 0.6-9 kHz filtered signal. Because we noticed that during some recording sessions the electrode seemed to have moved (e.g., due to movement of the monkey), we visually inspected the stability of each recording by investigating the stimulus aligned firing rates, MUAe and their baseline (−500 to −50 ms) energy across all trials and channels. With energy (E) defined as:

$$E = \int_i^t V(i)^2$$

where t is the number of time points in the vector (V) representing the single-trial histogram or MUAe. We selected the largest continuous time window that showed stable activity across all V1 & V4 channels.

In addition to selecting trials from stable periods, we selected channels for further processing that were determined to be in gray matter. Using current source density (CSD), we investigated on which channels currents were entering (sinks) and exiting (sources) cortical tissue, which allowed us to determine the relative recording depth compared to the known cortical anatomy (Schroeder et al.,

1991, 1998). The CSD profile can be calculated according to the finite difference approximation, taking the inverse of the second spatial derivative of the stimulus-evoked voltage potential φ, defined by:

$$CSD(x) = \frac{\varphi(x+h) - 2\varphi(x) + \varphi(x-h)}{h^2},$$

where x is the depth at which the CSD is calculated and h the electrode spacing (150 μm). We used the iCSD toolbox (Pettersen et al., 2006) to compute the CSD. With this toolbox we used a spline fitting method to interpolate φ smoothly between electrode contacts. We used a diameter of cortical columns of 500 μm (Mountcastle, 1957), and tissue conductance of 0.4 Sm^{-1} (Logothetis et al., 2007).

To aid determination of recording depth, we computed the signal-to-noise ratio (SNR), the response latencies to stimulus onset for each channel and the receptive field (RF) estimation (see below). SNR was computed as:

$$SNR = \frac{Signal - Noise}{\sigma_{noise}}$$

with *Signal* defined as the average MUAe amplitude in one of eight 50 ms time windows, from 30 to 80 ms, in 10 ms steps, to 100 to 150 ms after stimulus onset, and *Noise* as the average MUAe amplitude during the baseline period (200 to 50 ms) before stimulus onset. SNR in at least one of these eight estimates was required to be higher than 3 for a channel to be included for further analyses.

We computed the response latency to stimulus onset for each channel according to the method described by Roelfsema et al. (2007). We fitted the visual response as a combination of an exponentially modified Gaussian and a cumulative Gaussian using a non-linear least-squares fitting procedure (function lsqcurvefit) to the average MUAe time course. There are two assumptions implicit in this method. First, the onset latency has a Gaussian distribution across trials and across neurons that contribute to the MUAe, and second, that (part of) the response dissipates exponentially. The visual response y across time t was modeled as:

$$y(t) = d \cdot Exp\left(\mu\alpha + 0.5\sigma^2\alpha^2 - \alpha t\right) \cdot G\left(t, u + \sigma^2\alpha, \sigma\right) + c \cdot G(t, \mu, \sigma),$$

where μ is the mean, σ is the standard deviation, α^{-1} is the time constant of the dissipation, $G(t, \mu, \sigma)$ is a cumulative Gaussian, and c and d are the factors scaling the non-dissipating and dissipating modulation of the visual response. The response latency was defined as the time point where $y(t)$ reached 33% of the maximum of the earliest peak, the first Gaussian (Roelfsema et al., 2007; Self et al., 2013). Data were aligned to the earliest current sink, the presumed thalamic input layer (L4); channels were excluded if they were > 1 mm more superficial or > 0.75 mm deeper than this layer.

Receptive field estimation

Offline RFs were determined for each channel via reverse correlation of the MUAe signal (see above for MUAe computation) to stimuli (0.5 – 2° black squares) presented on a 9 × 12 grid (Gieselmann and Thiele, 2008). The stimulus-response map was converted to z-scores, after which the RF for each channel was indicated by a contour (thresholded at a z-score of 3) surrounding the peak activity. These z-scored maps were averaged across all channels for each area (the population average z-score was computed using Stouffer's Z-score method according to $Z = \sum_{i=1}^{k} Z_i / \sqrt{k}$, with k as the number of channels, after which we determined the overlap and separation between the V1 and V4 RFs (Figures 1B and 1C).

Bipolar re-referencing

To ensure that global signals, common to multiple channels, did not affect our LFP and spectral analyses (see below), we re-referenced our LFP signals according to the bipolar derivation. Bipolar re-referenced LFP signals (LFPb) were computed by taking the difference between two neighboring channels.

Attentional modulation

The effect of selective attention on neural activity was computed via an attention modulation index (*attMI*), defined as:

$$attMI = \frac{A_{RF} - A_{out}}{A_{RF} + A_{out}}$$

with A_{RF} as the neural activity when attention was directed toward the RF, and A_{out} the activity when attention was directed away from the RF. This index ranges from -1 to 1, with zero indicating no attentional modulation and with positive (negative) values indicating higher (lower) activity when attention was directed toward the RF.

Hidden Markov Model

To quantify On-Off dynamics in V1 and V4, we fit a Hidden Markov Model (HMM) to the population activity (MUA) across all laminae. We fit the HMM both to activity from each individual area, following the procedures described by Engel et al. (2016), as well as to the activity from both areas simultaneously.

Our HMM assumes that spike counts on the recorded channels can be well characterized as a doubly-stochastic process, of which the parameters can be accurately estimated (Rabiner, 1989). In this study, spike counts on each channel are assumed to be produced

by a Poisson process with different (constant) mean rates during On or Off phases of the underlying 'hidden' (latent) process s common to all channels that we need to infer (Engel et al., 2016). The mean firing rate on each channel j in phase s is defined by entry λ_j^s in the emission matrix Λ. The transition matrix P gives the probabilities of transitioning between these latent phases. In the transition matrix P, each entry indicates the probability of transitioning between two specific phases. For instance, P_{11} indicates the probability of transitioning from $s=0$ to $s=0$ (remaining in the Off phase), whereas P_{12} indicates the probability of transitioning from $s = 0$ to $s = 1$, more formally: $P_{11} = P_{off} = P(s_{t+1} = 0|s_t = 0)$, $P_{11} = P_{off} = P(s_{t+1} = 0|s_t = 0)$, $P_{12} = 1 - P_{off} = P(s_{t+1} = 1|s_t = 0)$. These probabilities do not depend on time: at any time step t, the probability of transitioning between phases depends only on the value of s at time t (s_t). The latent dynamics estimated by the HMM thus follow a discrete time series in which s_t summarizes all information before time t. For each channel, MUA was discretized by determining spike counts in 10 ms bins following each time t, with the probability of observing spike count n on channel j during phase s defined as

$$P(n|s) = \frac{\left(\lambda_j^s\right)^n}{n!} e^{-\lambda_j^s}$$

The full description of an HMM is given by the emission matrix Λ, transition matrix P and the probabilities π^0 that indicate the initial values s_0, in which $\pi_i^0 \equiv P(s_0 = i)$. These parameters were estimated using the Expectation Maximization (EM) algorithm (Bishop, 2006), maximizing the probability of observing the data given the model according to the Baum-Welch algorithm (Rabiner, 1989). Because the EM procedure can converge to a local maximum, rather than the global maximum, we repeated the EM procedure ten times with random parameter initializations and chose the model with the highest likelihood. Random values were drawn from Dirichlet distributions for π^0 and P, and from a uniform distribution between zero and twice the channel's mean firing rate for Λ. The EM procedure was terminated if the relative change, computed as $|new - original|/|original|$, in the log-likelihood was smaller than 10^{-3} and the change in the transition and emission matrix was smaller than 10^{-5}, or if it reached the maximum number of iterations (n = 500).

Once the optimal parameters were estimated, we used the Viterbi algorithm to determine the most likely latent trajectory for each individual trial. We applied the HMM separately to each attention condition. For every trial, we applied the HMM during multiple time periods of the task, during fixation and during the time window from 400 ms after cue onset to 30 ms after the first dimming event. For the behavioral analysis, we additionally analyzed the period up to 30 ms after the second dimming event for trials in which target dimming did not occur on the first dimming event, and for which the first distractor dimming was not inside the RFs.

To determine what number of latent phases best described the data, we fit HMMs with the number of phases ranging from 1 to 8, and used a four-fold cross-validation procedure to compute the leave-one-channel-out cross-validation error for each HMM (Engel et al., 2016). We fit the HMM to a randomly selected subset of 3/4 of the trials and computed the cross-validation error on the remaining 1/4 of trials. This procedure was repeated 4 times using a different 3/4 of trials for training and 1/4 of trials for testing the HMM. We computed the cross-validation error CV_{var} for each channel j across all trials K and time bins T as the difference between the actual and expected spike count according to:

$$CV_{var}[n_j] = \sum_{k=1}^{K} \sum_{t=1}^{T} \left(n_t^j - \lambda_j^{s_t}\right)^2$$

We normalized CV_{var} to the error in the 1-phase HMM, averaged across channels, cross-validations and conditions, and determined the difference in CV_{var} with each additional phase in the HMM. The normalized mean cross-validation error across each of the eight HMM models for all recordings is depicted in Figure S1. For most recordings, and for both V1 and V4, CV_{var} decreased with the addition of a second phase but did not decrease much further with additional phases. This allowed the identification of the elbow (kink) in this error plot as the model with two phases. We included areas/recordings for further analysis that revealed a reduction in cross-validation error of at least 10% with the addition of a second phase but did not decrease by more than 10% with additional phases. For a small subset of recordings, a three or a four-phase model fit the data best [V1: n = 7; V4: n = 1], suggesting that these recordings could contain states with systematically occurring nested fluctuations; these recordings were excluded from further analysis. In total, we found a reduction of > 10% in cross-validation error when fitting a 2-phase versus 1-phase model in 63 V1 (82.9%), and 72 V4 (92.3%) recordings; in 55 (77.5%) recordings we found evidence for a 2-phase model in both V1 and V4 (Figures S1A–S1D). For these recordings, epoch duration distributions closely followed an exponentially decaying function (Figure S1E), consistent with HMM assumptions, indicating that short epoch durations were most prevalent.

While the two-state HMM segments the data into discrete On and Off phases, our results do not depend on the assumption of discrete phases. Previous work showed that the On-Off dynamics can be also modeled with a continuous latent variable, in which case the inferred firing rates showed bimodality and dynamics consistent with that inferred by the HMM (Engel et al., 2016).

To investigate the across-area coordination of On-Off dynamics, we fit a 4-state HMM to V1 and V4 data simultaneously. Across these four states, both V1 and V4 could be in either an Off or On phase, with the states defined as: $V1_{off} - V4_{off}$ (state 1), $V1_{on} - V4_{off}$ (state 2), $V1_{off} - V4_{on}$ (state 3) and $V1_{on} - V4_{on}$ (state 4). This model was fit according to the same steps as the HMM applied to individual areas, with one exception. For each channel j, the emission rate λ was constrained to be the same across states for which this channel (area) was in the same phase. For example, rates were constrained for a V1 channel across state 1 and state 3, during which V1 was in an Off phase ($\lambda_j^{s=1} = \lambda_j^{s=3}$, $j \in V1$).

CellPress
OPEN ACCESS

Variance explained by the HMM

The amount of spiking variability captured by the HMM was determined using a two-fold cross-validation procedure in which we computed the fraction of variance explained (R^2) on a subset of data not used to fit the model (Engel et al., 2016). The HMM parameters were estimated on a random half of the trials (training trials) and used to decode the most likely On-Off state sequence on the remaining half of the trials (testing trials) not used for fitting the model.

For each channel j, the fraction of variance explained (R^2) in the MUA was computed over the total number of time bins N as

$$R^2 = 1 - \frac{Var_{res}[n_j]}{Var_{tot}[n_j]} = 1 - \frac{\sum_{t=1}^{N}\left(n_t^j - \lambda_j^{s_t}\right)^2}{\sum_{t=1}^{N}\left(n_t^j - \langle n_t^j \rangle\right)^2}$$

Where $Var_{tot}[n_j] = \frac{1}{N}\sum_{t=1}^{N}\left(n_t^j - \langle n_t^j \rangle\right)^2$ is the total variance of the spike-count data and $Var_{res}[n_j] = \frac{1}{N}\sum_{t=1}^{N}\left(n_t^j - \lambda_j^{s_t}\right)^2$ is the residual variance unaccounted for by the HMM.

As the HMM was fitted to MUA, we slightly altered the cross-validation procedure for SUA. Instead of fitted as a model parameter, SU firing rates were estimated as the mean rate during On and Off phases of the most likely latent state sequence on training trials, decoded from the MUA. These estimated On and Off firing rates were used in the above formula instead of $\lambda_j^{s_t}$ to compute R^2 on test trials.

We computed R^2 across different timescales (Figure S3B) by computing MUA and SUA spike-counts for 10 different window sizes (integration times) ranging from 50 to 500 ms in 50 ms steps. We followed the procedures described above except that the predicted spike-count was the average of the firing rates predicted by the HMM within each integration time bin.

In order to interpret the results from the cross-validation analysis, we computed the maximal explainable variance R^2_{max} given that the HMM assumptions hold true; that spikes are produced by a Poisson process where the mean rate switches between two levels corresponding to the On and Off phases, and that the latent sequence of On and Off epochs (and their corresponding firing rates) in each phase are known precisely (Engel et al., 2016). Under these assumptions, all variance due to rate fluctuations is eliminated by the precise knowledge of the mean firing rate in each time bin. Any residual variance of spike-count $Var_{res}[n]$ is therefore just the variance of the Poisson point-process, which is equal to its mean E_n. The maximal explainable variance R^2_{max} therefore directly relates to the Fano factor (FF), a measure of firing rate variability defined as the variance over the mean of the spike count, and can be computed as

$$R^2_{max} = 1 - \frac{E[n]}{Var_{tot}[n]} = 1 - \frac{1}{FF}$$

The R^2_{max} curves in Figure S3A depict the evaluation of this equation. In order to compute the average maximal explainable variance curves depicted in Figure S3C, we first computed FF for each unit and the accompanying R^2_{max} value using the specified integration window, and then we averaged across the population.

Dependence of On-Off dynamics on firing rates

Investigating cortical state requires the analysis of population activity, i.e., the pooled activity of many individual neurons. If one would record using probes with a high density and number of channels (e.g., Neuropixel, Jun et al., 2017), allowing recording of large numbers of single units, it would be feasible to determine the state of the population activity using only single unit activity. However, given the recording density of this dataset (16 electrode contacts with 150 μm spacing) accurately estimating network state based solely on single units is not feasible. We therefore opted to estimate population activity by progressively lowering thresholds until each channel had a firing rate of approximately 100 Hz (MUA100). To test whether the latent state estimation (the HMM fit) was contingent on this specific definition of population activity we also extracted MUA according to a different definition. We extracted MUA by progressively lowering spike extraction thresholds until approximately 20 Hz spiking activity was detected on each channel during fixation (spontaneous activity), in the 400 ms preceding stimulus onset (MUA20). We then fit the same HMM as described above and compared the cross-validation error and variance explained (R_2) between these two MUA definitions.

We found that MUA20 had a mean trial-averaged firing rate of approximately 33 Hz in the time period between 400 ms after cue onset until first dimming (Figure S4A). The correlation between R_2 values for MUA100 and MUA20 was highly significant for both V1 [r = 0.81, p = 10^{-146}] and V4 [r = 0.88, p = 10^{-224}] (Figure S4B). Despite this high correlation, we did find that R_2 was higher for MUA100 compared to MUA20, both for V1 and V4 [two-sided Wilcoxon signed rank test; V1: p < 10^{-82}; V4: p < 10^{-95}]. Together, these results show that the HMM fit is not contingent on the specific definition of population activity, but that the fit is better with higher firing rates.

We additionally investigated cross-validation error to determine for how many recordings a 2-phase model was most parsimonious. We found a reduction of > 10% in cross-validation error when fitting a 2-phase versus 1-phase model in 53 V1 (instead of 63), and 61 V4 (instead of 72) recordings (Figures S4C and S4D). Thus in addition to slightly lower R_2 values for MUA20 compared to MUA100, we also found that fewer recordings met our criteria to determine whether a 2-phase model fits the data best.

Figures S4E and S4F depict one example trial that reveals the difference in activity levels and HMM fit between MUA20 and MUA100. Although the overall estimated latent state sequence is highly similar, the HMM based on MUA20 missed shorter On or Off epochs that were found using MUA100. Thus, although estimating latent phases based on population activity with low firing rates is possible, using higher rates provides a better overall fit and might allow better classification of shorter state epochs.

Dependence of On-Off dynamics on task timings

As the task timings are drawn from uniform distributions, it is possible that On-Off dynamics are, in part, driven by a non-flat hazard function. To investigate this possibility, we computed the probability density function (PDF) of On-Off and Off-On transitions for attend RF and attend away conditions according to

$$v_t = \frac{c_t}{w_t}$$

with probability v of having transition count c for each time bin t with width w (100 ms bin width). Next, we computed an attention modulation index (*attMI*, see above) and tested whether it differed from zero for each time bin (Figure S5G).

Rate-matching control

We investigated whether the attentional modulation of On-Off dynamics could be confounded by higher trial-averaged firing rates in the attend RF condition. As longer On episode durations bring about higher trial-averaged firing rates, the HMM fit might account for higher trial-averaged firing rates by estimating longer On episodes. This possibility is unlikely, however, as the free parameters controlling episode durations (emission rates and transition possibilities) are fit for each condition independently. An additional possibility is that the ability to detect transitions may be impaired with lower spike counts, reducing sensitivity to transitions and thereby registering longer On episode durations during attend RF trials. We performed a rate-matching analysis to control for any potential confounding effects due to rate differences across attention conditions.

We equated the trial-averaged firing rates across the three attention conditions by randomly deleting subsets of spikes from conditions with higher rates (Figures S5C and S5D). We then fit the HMM to the rate-matched data and estimated the durations of On and Off episodes as well as the fraction of time spent in either phase (Figure S5E). In the rate-matched data, the increased On-phase duration in V1 and V4 and the increased Off phase duration in V1 in the attend RF condition were preserved. However, after rate-matching, we additionally found increased Off phase durations in V4. As the increased fraction of time spent in an On phase in the attend RF condition was also preserved in the rate-matched data (Figure S5F), this change in Off phase duration in V4 did not alter the time spent in either phase. These results show that the attentional modulation of On episode durations and the time spent in an On phase are not artifacts of higher mean firing rates during attention conditions.

On-Off dynamics and behavioral performance

To determine the effect of On-Off dynamics and their across-area coordination on behavioral performance, we investigated whether the On/Off phase of population activity at the time of target dimming influenced reaction times (RT). To this end, we averaged, for each recording, the RT across all trials that ended in the same phase. We subsequently tested for a relationship between On/Off phase and RT across recordings.

Cross correlation

The temporal relationship between On-Off time series and transitions, microsaccade onset times and activity in V1, V4 and FEF were investigated using cross-correlations. The cross-correlations based on HMM time series (CC_{HMM}) were calculated using the function xcorr in MATLAB, according to:

$$CC_{HMM}(\tau) = \frac{1}{M}\sum_{m=1}^{M} \frac{\sum_{t=1}^{T} x(t)y(t+\tau)}{\sqrt{\sum_{t=1}^{T}|x(t)|^2 \cdot \sum_{t=1}^{T}|y(t)|^2}}$$

where M is the number of trials, T is the number of discrete time bins, x and y the mean-subtracted On-Off time series in V1 and V4 as determined by the HMM, and τ the time lag. Here, the numerator indicates the cross-covariance, which is normalized (the denominator) such that the autocorrelation for each time series at zero lag is 1. This procedure normalized CC_{HMM} such that correlation coefficients were obtained. We furthermore subtracted the shuffle predictor $CC_{shuffle}$ from CC_{HMM} to remove any task-related (event-locked) correlations between x and y. $CC_{shuffle}$ was computed by shuffling y trials.

Cross-correlations (CC) between state transitions and microsaccade onset times were computed in the same way but for a different normalization (denominator) factor. Here we normalized by the number of microsaccades, resulting CC to be of the order of coincidences of state transitions per microsaccade.

To investigate the neural activity around the time of On-Off transitions, we computed the transition-triggered average (TTA). The TTA was estimated by computing the cross covariance (the numerator), divided by the number of transitions for each channel (denominator). Again, we subtracted the shuffle predictor to remove any task-related correlations.

CellPress
OPEN ACCESS

Power estimation

We estimated the power spectra of the bipolar re-referenced LFP using a custom multitaper approach based on the Chronux toolbox (Bokil et al., 2010). We estimated the power separately for On and Off states determined by the HMM using only epochs that lasted longer than 250 ms. Because epoch durations were variable, we zero-padded each segment to the next highest power of 2 (1024 time points), ensuring we could extract the same frequencies for each segment. This approach gave us a half bandwidth (W) of approximately 3.97 Hz, according to $W = (K+1)/2T$, with K being the number of data tapers ($K = 7$) and T the length of the time window in seconds. Frequencies were estimated from 4 to 200 Hz.

Microsaccades and their relation to On-Off dynamics

Neural activity in visual cortex is influenced by the small fixational eye movements termed microsaccades (Bair and O'Keefe, 1998; Leopold and Logothetis, 1998). Attentional modulation of neural activity in areas V4 and IT has furthermore been found to depend on whether microsaccades were directed toward the attended stimulus, as indicated by a spatial cue (Lowet et al., 2018). It is therefore likely that On-Off dynamics are also influenced by microsaccades. We investigated the relationship between On-Off dynamics and microsaccades in order to determine whether microsaccades could account for the modulation of On-Off dynamics during attention.

We detected microsaccades by using the algorithm developed by Engbert and Kliegl (2003). We converted eye position to velocity and low-pass filtered the velocity traces (Goettker et al., 2018; Lowet et al., 2018) at 20 Hz using a 2nd order Butterworth filter. An eye movement was classified as a microsaccade if the velocity is larger than a threshold for at least three consecutive time points. The threshold is set to six times the median estimator, given by: $\sqrt{median(x^2) - median(x)^2}$, where x is the eye position channel. Thus, the threshold is determined for each single trial. The use of the median estimator ensured that microsaccade detection is relatively robust to different levels of noise.

We detected microsaccades with an average amplitude of 0.50 ± 0.28° (mean ± standard deviation) and velocity of 18.55 ± 8.44 (Figure S6A). In line with previous studies (Engbert and Kliegl, 2003; Martinez-Conde et al., 2009, 2013), 94.6% of detected microsaccades were smaller than 1° and they occurred on approximately 57 ± 3% (mean ± SE) of trials (Figure S6B) with a rate of approximately 0.9 ± 0.08 Hz (Figure S6C). There was no difference in the proportion of trials on which we detected microsaccades [repeated-measures ANOVA; $F(2) = 2.75$, $p = 0.07$] or in the microsaccade rate [repeated-measures ANOVA; $F(2) = 1.29$, $p = 0.28$] across the three attention conditions. On trials where we detected multiple microsaccades, the inter-microsaccade interval was 307 ± 219 ms (mean ± standard deviation) (Figure S6D).

To control for the possibility that we did not detect all microsaccades, we tested whether the direction of gaze differed across attention conditions on trials without microsaccades. We computed average gaze positions between 400 ms after cue onset until first-dimming and rotated these gaze positions such that the attended stimulus location aligned with the horizontal axis. We then fit a 2D Gaussian to the gaze positions across trials for each recording and tested the horizontal offset from zero (Figure S6E). Although there was a small tendency to direct gaze away from the attended stimulus on attend RF trials and toward the attended stimulus on attend away 2 trials [two-sided Wilcoxon signed rank test, attend RF: $p = 0.02$; attend away 1: $p = 0.97$; attend away 2: $p = 0.04$], neither of these effects survived FDR correction for multiple comparisons. Thus, gaze effectively did not deviate from fixation on trials without microsaccades for any of the attention conditions.

We investigated the relationship between microsaccades and On-Off dynamics by computing cross-correlations (see above) between microsaccade times and the times of On-Off and Off-On transitions (Figure S6F). The cross-correlation between the times of microsaccades and Off-to-On transitions exhibited a positive peak at approximately 60 ms and 140 ms time-lag for V1 and V4, respectively, indicating that the probability of transition from Off to On phase was enhanced following a microsaccade. In V4, the cross-correlation between the times of microsaccades and On-to-Off transitions exhibited a negative peak at approximately 140 ms time-lag, indicating that transitions from On to Off phase were less likely to occur following a microsaccade.

We next investigated the possibility that attentional modulation of On-Off dynamics could result from changes in the direction of microsaccades between attention and control conditions. We compared the microsaccade frequency between attend RF and attend away conditions across microsaccade directions aligned to the RF location, corresponding to 0° in Figure S6G. Although the relative frequency of microsaccades between attention and control conditions differed across directions for each subject [chi-square test, $p < 10^{-9}$], the pattern of this effect was markedly different across subjects. Whereas microsaccades were more frequently directed toward the location of the RF for monkey T, they were more frequently directed in the opposite direction for monkey W, and for monkey J they were more frequently directed toward the stimulus at 240°, corresponding to the attend away 2 condition (chi-squared residuals test at 0.05 significance level with Bonferroni correction).

Next we tested whether the direction of microsaccades influenced On-Off dynamics. As the microsaccade rate during attend RF conditions was not increased compared to control conditions (Figure S6C), directional biases in microsaccade frequency should only contribute to the observed increased On-episode durations if those biases coincide with increases in the Off-to-On transition rate following microsaccades in the bias directions, or with decreases in the On-to-Off transition rate following microsaccades in the bias directions. However, the fraction of microsaccades followed by Off-to-On transitions was not significantly different across microsaccade directions for either V1 or V4 [Figure S6H, chi-square test, V1: $p = 0.99$; V4: $p = 0.35$]. The fraction of microsaccades followed by On-to-Off transitions was also not significantly different across microsaccade directions [chi-square test, V1: $p = 0.99$; V4: $p = 0.22$]. Thus the rate of On-Off transitions was independent of microsaccade directions and changes in the microsaccade rate and direction cannot explain the increase of On-episode durations during attention.

Finally, we further ruled out whether microsaccades brought about the increased On-epoch duration or the top-down state coordination between V1 and V4 by repeating all analyses for the trials in which no microsaccades occurred during the analyzed time period. Although this procedure left us with fewer trials, the pattern of the results was unchanged. On-episode durations were increased when attention was directed toward the RF for both V1 and V4 [two-sided Wilcoxon signed rank test, V1: $p < 10^{-7}$; V4: $p < 10^{-7}$] and Off-episode durations were increased in V1 but not V4 [two-sided Wilcoxon signed rank test, V1: $p < 10^{-6}$; V4: $p = 0.95$] (Figure S6). Similarly, the exclusion of trials in which microsaccades occurred did not alter the pattern of phase coordination between V1 and V4. In the time period between 400 ms after cue onset and first-dimming, the cross-correlation between V1 and V4 latent state was skewed toward negative values [two-sided Wilcoxon signed rank test, $p = 0.002$], indicating that V4 state transitions occur before those in V1. Thus, neither the increase in On-episode durations during attention nor the top-down coordination of latent states across V1 and V4 were due to microsaccades.

QUANTIFICATION AND STATISTICAL ANALYSIS

To determine whether there were significant differences between attention conditions or HMM states (e.g., in firing rate or epoch duration) we made use of multiple statistical methods. We used (paired sample) Wilcoxon signed rank tests whenever a comparison was made between two conditions (e.g., attend RF versus attend away), or to test whether a distribution was significantly different from zero. When a comparison involved multiple conditions, or multiple factors (e.g., attention and state), we used linear mixed effect models to test for main effects of each condition/factor and interaction effects between factors. These factors were defined as fixed effects and we included random intercepts for each recording as random effects, accounting for the repeated-measurements within each recording. Specifically, we modeled RT as a linear combination of attention condition (Att) and HMM state coefficients, as well as their interaction:

$$RT \sim \beta_0 + \beta_1 Att + \beta_2 HMM + \beta_3 Att \cdot HMM$$

We used false discovery rate (FDR) to correct for multiple comparisons (Benjamini and Yekutieli, 2001). Error bars in all figures indicate the standard error of the mean (SEM).

Neuron, Volume *109*

Supplemental information

Top-down coordination of local cortical state during selective attention

Jochem van Kempen, Marc A. Gieselmann, Michael Boyd, Nicholas A. Steinmetz, Tirin Moore, Tatiana A. Engel, and Alexander Thiele

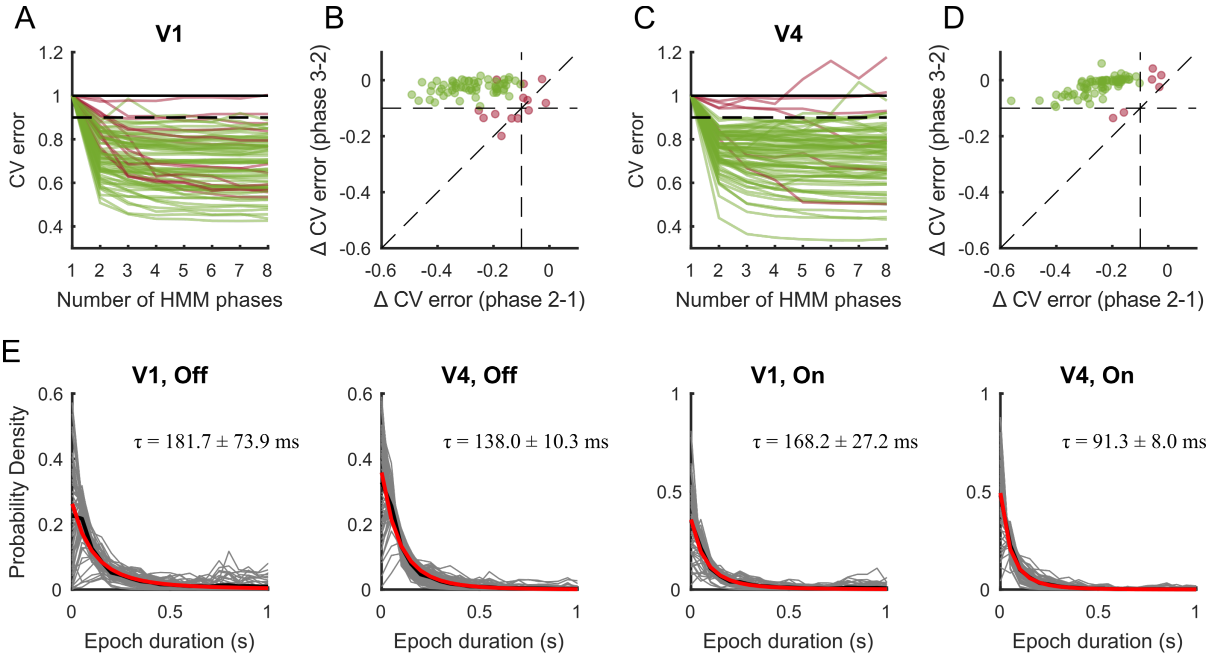

Figure S1. Determining the number of HMM phases and their epoch durations in V1 and V4 MUA, related to STAR Methods. (A) Cross validation (CV) error plotted against the number of phases in each HMM for V1. (B) The difference in cross validation error between the 1-phase and 2-phase model, plotted against the difference between the 2-phase and 3-phase model for V1. Most recordings show a large reduction in cross-validation error with the addition of a second phase and only marginal changes with additional phases. Green (red) lines and markers indicate the recordings included (excluded) for further analysis. (C-D) Same conventions as (A-B) but for V4. (E) Distributions of Off and On episode durations overlaid by the exponential distributions with the decay constant set by the HMM transition probabilities (red, $N(t) = N_0 e^{-t/\tau}$, where N_0 is the normalisation constant, and τ is the decay time-constant computed for each recording and phase). A good match for these models indicates that On-Off dynamics were not driven by an oscillatory phenomenon. Grey and thick black lines indicate individual recordings and their mean, respectively. Data are represented as mean ± SEM across recordings.

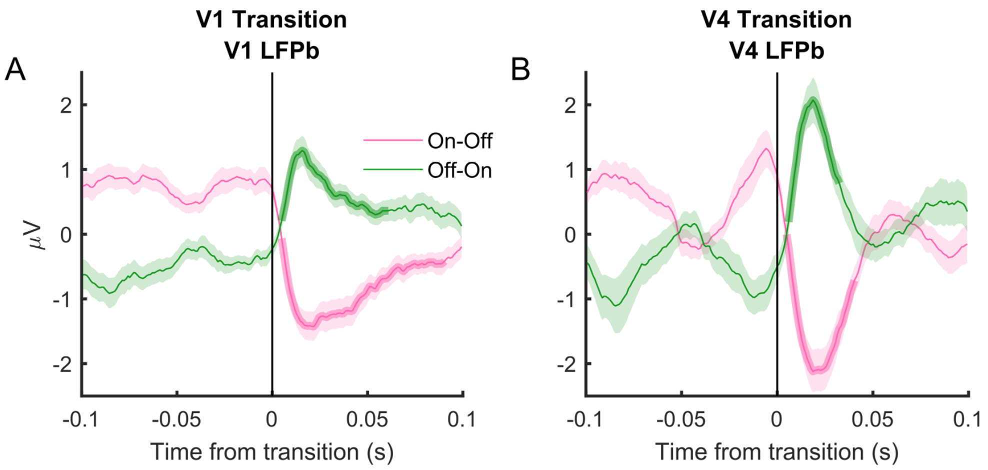

Figure S2. Relationship between local field potentials (LFPs) and On-Off transitions, related to STAR Methods. Bipolar re-referenced LFP activity aligned to state transitions for V1 (A) and V4 (B), averaged across channels and recordings. Only epochs without transitions preceding or following the alignment transition within 100 ms were included. Thick green and pink lines indicate the times the activity was higher (green) or lower (pink) than the average activity (FDR-corrected, one-sided Wilcoxon signed rank test). Data are represented as mean ± SEM across recordings.

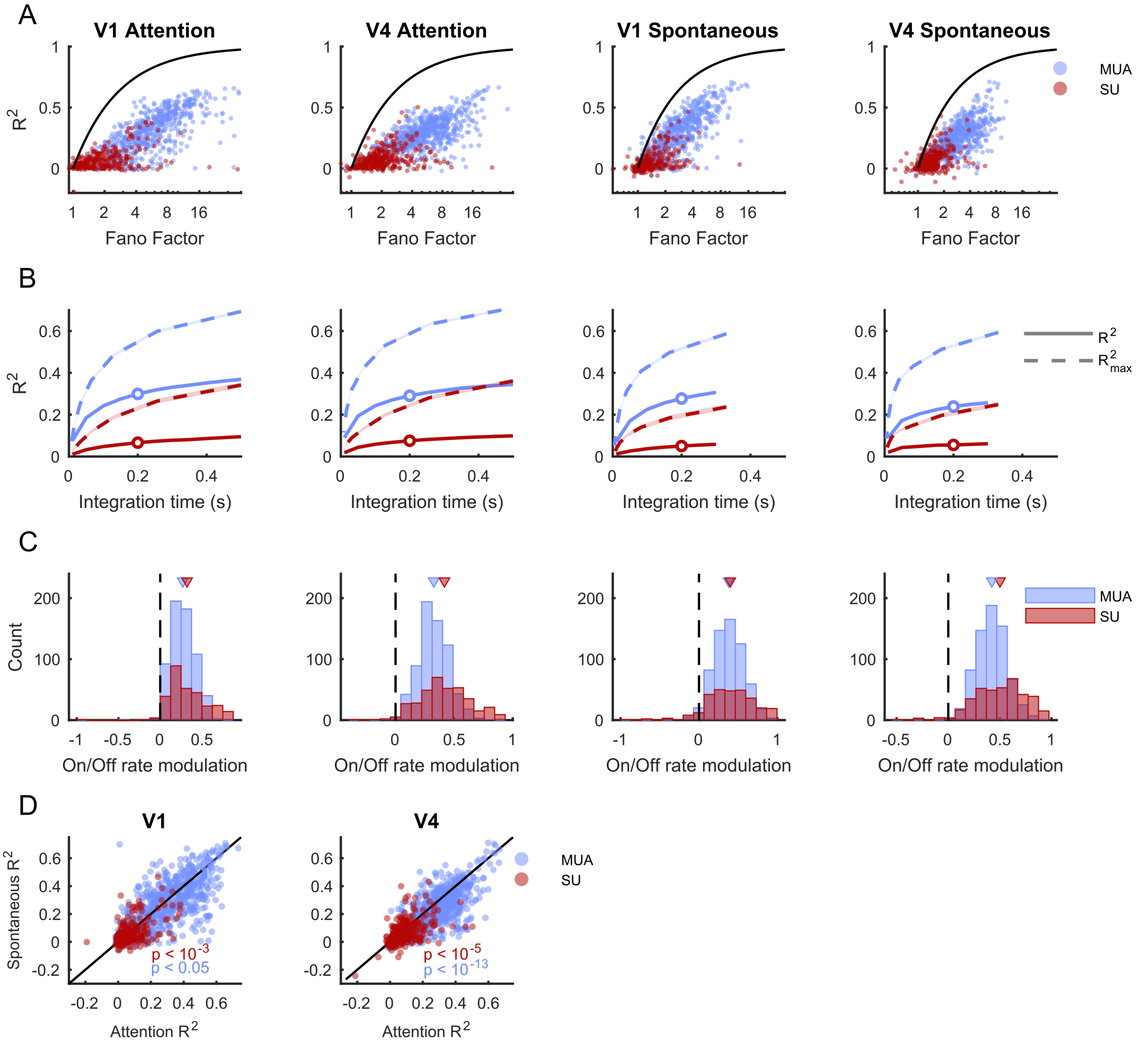

Figure S3. Variance explained by On-Off transitions, related to STAR Methods. (A) Scatter plot of the variance explained by the HMM versus the spike-count variability measured by the Fano factor for single- (SU, red dots) and multi-units (MU, blue dots). Each dot represents the average across all trials of one recording. Solid black line depicts the maximal explainable variance for each Fano factor value. (B) Population-average of the variance explained by the HMM as a function of the integration time window for single- (SU, red line) and multi-units (MU, blue line). Dashed lines depict the corresponding maximal explainable variance. White circles indicate 200 ms integration time window that was used in panel A. Error bars are ±SEM. (C) Distribution of the On/Off firing rate modulation index ([ron − roff]/[ron + roff]) for single- (SU, red) and multi-units (MU, blue). From left to right is plotted V1 during attention, V4 during attention, V1 during fixation (spontaneous activity) and V4 during fixation. (D) Variance explained in spontaneous activity plotted against that during attention for V1 (left) and V4 (right). Statistics: two-sided Wilcoxon signed rank tests.

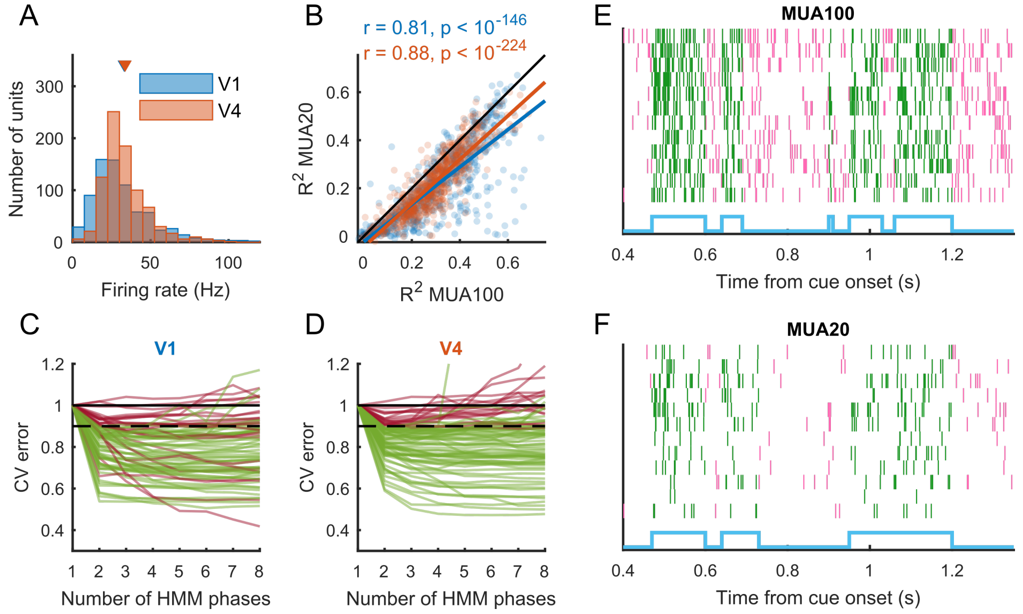

Figure S4. MUA definition control analysis, related to STAR Methods. (A) Trial-averaged firing rate in the period between 400 ms after cue onset until first dimming for MUA extracted based on a 20 Hz firing rate during the spontaneous period. (B) Variance explained (R^2) by the HMM for the original 100 Hz data (MUA100) plotted against R^2 for MUA extracted based on spontaneous activity (MUA20) for V1 (blue) and V4 (red). Each marker depicts one channel. The HMM fit is highly similar, as shown by their strong correlation indicated by the blue and red line. Statistics: Pearson correlation. As most markers fall below the black unity line, R^2 is higher for MUA100. (C-D) Cross validation (CV) error for MUA extracted based on spontaneous activity plotted against the number of phases in each HMM for V1 (C) and V4 (D). Compare to Figure S1A & C. (E-F) Example trial across-laminar raster plot and the corresponding latent state (blue) for MUA100 (E) and for MUA20 (F).

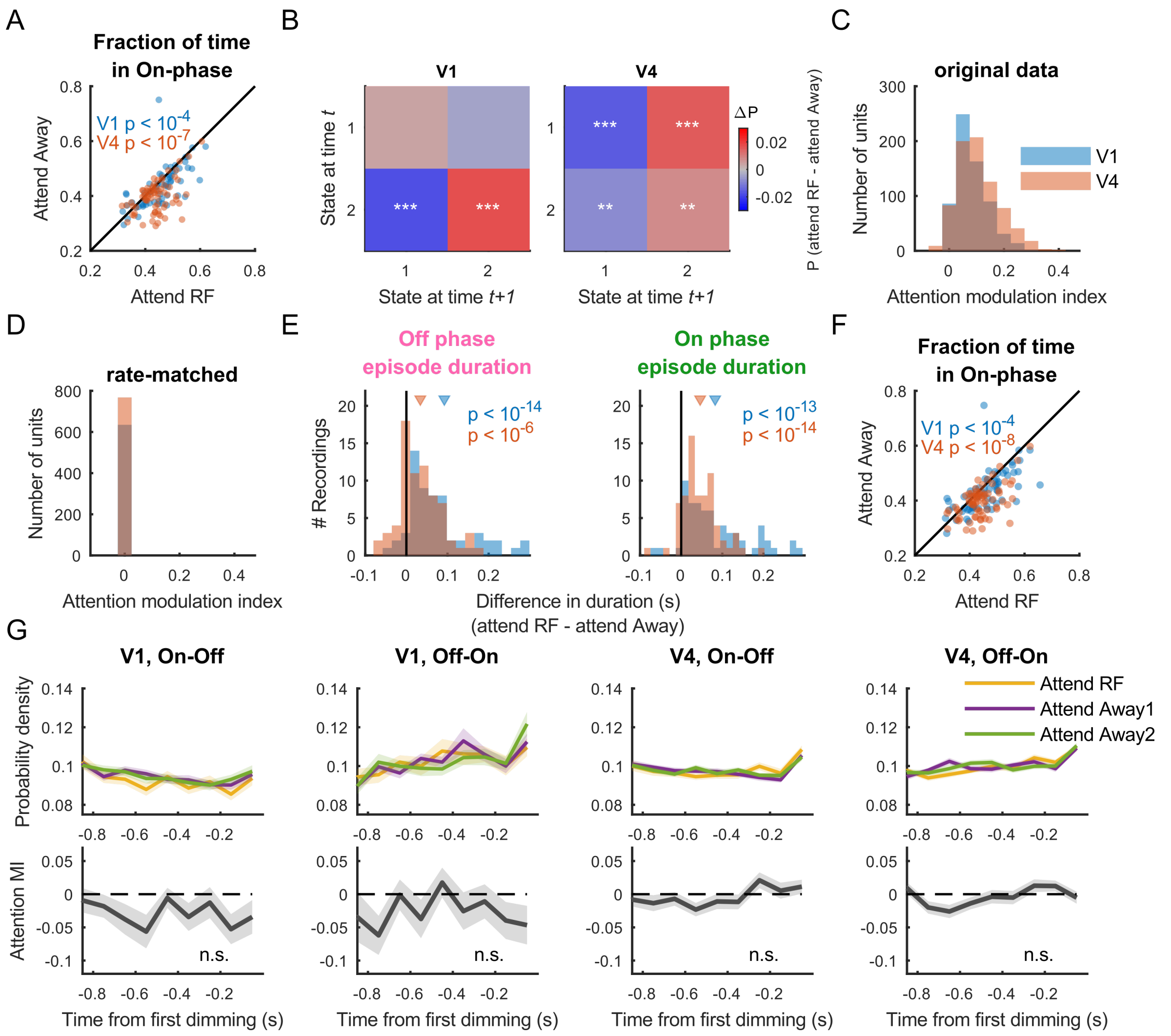

Figure S5. Attentional modulation of HMM parameters and rate-matching control, related to Figure 1 and STAR Methods. (A) The fraction of time spent in an On phase is increased when attention is directed towards the RFs. (B) Attentional influence on HMM transition probabilities. Shown is the difference between transition matrices (attend RF – attend Away). (C-F) Rate-matching control analysis. (C) Distribution of attention modulation index of the mean firing rate of multi-units for V1 (blue) and V4 (red). Trial-averaged mean firing rate was higher in attend RF conditions compared to attend away conditions. (D) As in C, but after rate-matching across attention conditions. (E) Distribution of the difference ($\tau_{Attend\ RF} - \tau_{Attend\ away}$) in average duration of the On (right panel) and Off (left panel) episodes between attend RF and attend away conditions after rate matching. The increase in On episode durations is preserved in the rate-matched data, thus this effect was not an artifact of higher mean firing rates during attention conditions. (F) The increased fraction of time spent in an On phase when attention is directed towards the RFs is preserved in the rate-matched data. (G) Transition probability density function (PDF) for phase transitions aligned to the first-dimming event (top) and its attentional modulation index

(MI, bottom; $MI = (Attend_{RF} - Attend_{Away})/(Attend_{RF} + Attend_{Away})$). From left to right: V1 On-Off transitions, V1 Off-On transitions, V4 On-Off transitions and V4 Off-On transitions. Data are represented as mean ± SEM across recordings. As no attention MI time bin deviated from zero, transition PDFs did not differ across attention conditions. Statistics: two-sided Wilcoxon signed rank tests; *, **, ***, indicate FDR corrected significance levels of $p < 0.05$, $p < 0.01$ and $p < 0.001$, respectively.

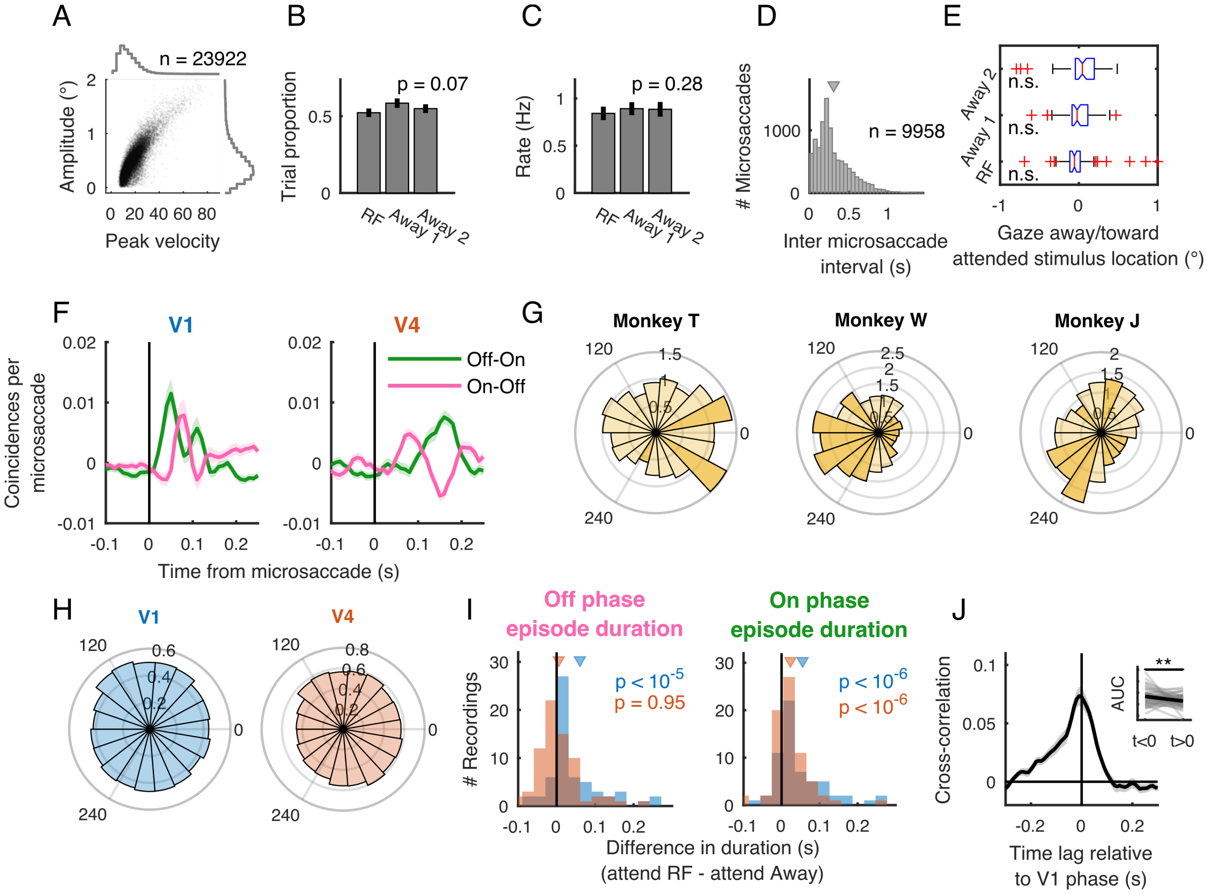

Figure S6. Relationship between microsaccades and On-Off transitions, related to Figure 1, Figure 2 and STAR Methods. (A) Microsaccade amplitude versus velocity plot. The histograms along the top (right) indicate the distribution of velocities (amplitudes). (B) Proportion of trials with microsaccades across attention conditions. (C) Microsaccade rate across attention conditions. (D) Distribution of inter-microsaccade-intervals. (E) Average gaze on trials without microsaccades relative to the location of the attended stimulus. Raw gaze locations were rotated such that the attended stimulus location was aligned to 0°, the x-direction therefore indicates gaze towards or away from the stimulus. (F) Cross-correlation of On-Off transitions in V1 (left) and V4 (right) triggered to microsaccade onset. (G) Relative microsaccade frequency between attention and control conditions across microsaccade directions, aligned to the RF location for each subject. The relative frequency is computed for each direction (in 20° bins) as the ratio of the number of microsaccades towards that direction in attention over control conditions. 0° corresponds to the RF location, which aligns with the attended stimulus in the attend RF condition. 120 and 240° correspond to the attend away stimulus locations. Directions with significantly higher relative microsaccade frequency are highlighted with darker color-fill (chi-squared residuals test at 0.05 significance level with Bonferroni correction). Microsaccades were not systematically directed towards or away from the RF location across subjects. (H) Fraction of microsaccades that were followed by Off-On transitions across

microsaccade directions, aligned to the RF location. The fraction of microsaccades followed by transitions did not significantly vary across microsaccade directions (chi-squared test, V1: $P=0.99$; V4: $P=0.38$). (I) The increase of On-episode durations in V1 (blue) and V4 (red), and the increase in Off-episode durations in V1 is preserved after exclusion of trials in which microsaccades occurred. (J) Cross-correlation between time series of On-Off dynamics in V1 and V4 after exclusion of trials in which microsaccades occurred. Statistics: two-sided Wilcoxon signed rank test (panel E, I and J) and a repeated measures ANOVA (panel B and C). Data are represented as mean ± SEM across recordings; *, ** and *** indicate significance levels of $p < 0.05$, $p < 0.01$ and $p < 0.001$ respectively.

	**Red**	**Green**	**Blue**
**Monkey 2 & 3, and monkey 1 (n=4)**	a. [220 0 0] – 12.8<br>b. [140 0 0] – 4.2	a. [0 135 0] – 12.9<br>b. [0 90 0] – 4.6	a. [60 60 255] – 12.2<br>b. [30 30 180] – 4.6
**Monkey 1 (n=1)**	b. [170 0 0] –6.7	b. [0 105 0] – 6.4	b. [37 37 210] – 6.6
**Monkey 1 (n=1)**	b. [175 0 0] –7.2	b. [0 105 0] – 6.4	b. [40 40 220] – 7.7
**Monkey 1 (n=8)**	b. [180 0 0] –7.7	b. [0 110 0] – 7.3	b. [40 40 220] – 7.7

Table S1. Color values used for the three colored gratings across recording sessions and subjects, related to STAR Methods. Color values are indicated as [RGB] – luminance (cd/m^2). a = Undimmed values, b = Dimmed values. For monkey 1, we used a variety of dimmed values across recordings.
